
JOURNAL INFORMATION
==============================
NLM Title Abbreviation: J Child Orthop
Journal ID: J Child Orthop
NLM Journal ID: 101313582

Journal of Children's Orthopaedics

ISSN: 1863-2521
EISSN: 1863-2548
Publisher: SAGE Publications

ARTICLE INFORMATION
==============================
PMCID: PMC4252273
PMID: 25258053
DOI: 10.1007/s11832-014-0608-x
Article ID: 608
Article version: 1
Subjects: Abstracts

28th Annual Meeting of the Children’s Orthopaedics, Bonn, 15–16 March 2014

Electronic publication date: 2014 Sep 26
Print publication date: 2014 Dec
Volume: 8
Issue: 6
First page: 521
Last page: 546
Copyright: © The Author(s) 2014
License: The abstracts are distributed under the terms of the Creative Commons Attribution License which permits any use, distribution, and reproduction in any medium, provided the original author(s) and the source are credited.
License URL: https://creativecommons.org/licenses/by/4.0/

issue-copyright-statement: © The Author(s) 2014

==============================

### Contents

Undesirable Treatment Results .................................................. 522
- Posttraumatic Stiff Elbow in Children: What Outcomes Should be Expected After Surgical Treatment? .................................. 522
- Recurrence of Axial Malalignment After Removal of Temporary Hemiepiphysiodesis ............................................................ 522
- Spastic Gastrocnemius Fascicles Do Not Lengthen When Braced with AFOs........................................................................... 523
- Growth Disturbances of the Distal Tibia After Circumferential Release for Idiopathic Clubfeet: 10 Years Follow-up ......... 523

Torsionsmalalignement.............................................................. 524
- Classification of Rotational Deformities of the Legs in Children and Adolescents and Their Associated Gait Patterns .......... 524
- Causes of Intoeing: Gait Mechanics in Patients with Reduced Tibial Torsion Versus Patients with Increased Femoral Torsion..... 524
- Does Rotational Malalignment Cause Knee Pain? Comparison of Gait Between Symptomatic and Asymptomatic Children and Adolescents with Rotational Problems of the Legs...... 524

Paediatric Orthopaedic Problems in the Developing World ..... 525
- Treatment of the Neglected Clubfoot in the Third World, a Follow up of 6 Years ....................................................................... 525
- Pediatric Orthopedic Work in Resource Deprived Countries. Transfer of Knowledge by Active Tuition in Tanzania. Example: Blount's Disease ................................................................. 525
- Imminent Loss of Extremity Due to Chronic Osteomyelitis and Soft Tissue Infection in Children ................................ 526

Hip/Upper Extremity/Free Topics ............................................ 526
- Combined Pelvic and Femoral Osteotomy in Patients with Perthes' disease: Clinical and Radiological Outcome at Skeletal maturity ....................................................................................... 526
- Quality of Life After Intense Surgery in Patients with Perthes-Disease ................................................................................. 526
- Growth and Deformity after In Situ Fixation of Slipped Capital Femoral Epiphysis .............................................................. 527
- Arthroscopic Treatment of Septic Arthritis of the Hip in Children ............................................................................... 527
- Operative Treatment of the Hands and Feet in Apert Syndrome ............................................................................ 527
- 5 Year Outcome of Ulnar Osteotomy and External Fixation for Radial Head Dislocation ............................................... 528
- Management of the Tibia in Osteogenesis Imperfect Congenita (OIC) with Special Emphasis on the Choice of Implant, Surveillance and Implant related Complications ............................ 528
- Ultrasonography of Osteochondritis Dissecans of the Knee Joint 528

Neuroorthopaedics ..................................................................... 529
- Functional Monitoring of the Upper Extremity in Children with Spastic Unilateral Cerebral Palsy................................ 529
- Brain Plasticity Related to Hand Motor Intensive Training in Youths with Cerebral Palsy............................................. 529
- Distal Femoral Extension Osteotomy in Children with Cerebral Palsy..................................................................................... 529

Foot and Lower Leg................................................................... 530
- Pressure Distribution of Children with Clubfoot Treated with the Ponseti Method and Operative Methods............... 530
- Congenital Vertical Talus: In Search of the Best Treatment Algorithm............................................................................... 530
- A Prospective Radiological Study for Treatment of Iuvenile Flexible Flatfoot: Effect of the Calcaneus Stop Screw to the Heel Valgus .................................................................................. 531
- Total Tibial Agenesia: Therapeutic Options and Results of Treatment .............................................................................. 531
- Long-Time Consequences of Avulsion Fractures of the Proximal Tibia in Adolescence........................................................... 531
- Accordance of Preoperative Magnetic Resonance Imaging (MRI) and Arthroscopic Findings in Juvenile Osteochondritis Dissecans (OCD) .................................................................................. 532

Spine ........................................................................................... 532
- Long-term Follow-up after Posterior Hemivertebra Resection with Transpedicular Instrumentation in Young Children with Congenital Scoliosis.................................................................... 532
- Results of 10 Years with the Growing Rod System in the Treatment of Scoliosis ................................................................. 533
- Case Series of Five Children with Symptomatic Intraspinal Exostoses ................................................................................. 533

Neuroorthopaedics 2.................................................................. 533
- Kinematics During Stair Climbing in Cerebral Palsy ......... 533
- Functional Improvement After Selective Dorsal Rhizotomy in Children with Bilateral Spastic Cerebral Palsy .............. 534
- Effect of Selective Dorsal Rhizotomy in Patients with Bilateral Cerebral Palsy GMFCS I/II ................................................. 534
- Children with Spastic Hemiplegia and Bilateral Involvement: Do They Require Uni or Bilateral Management?...................... 535

Poster-Presentation 14.03. 15.03 ... 535
The Surgical Treatment of Non-idiopathic Spinal Deformity: Better Results in Younger Patients? ... 535
Osteoblastoma in the Paediatric Spine: Surgical Management ... 535
Treatment of Juvenile Foot Deformities with External Fixator ... 536
Spectrum and incidence of Anomalies of foot in Fibular Hemimelia ... 536
Pedobarographic Results of Clubfeet After Peritalar Release Surgery ... 537
Flexor Hallucis Longus Dysfunction: an Underestimated Problem? ... 537
Pes equinus in Young Women: Psychogen Reasons have to be Considered ... 537
Late Diagnosis in Tarsal Coalition ... 538
Hip Dysplasia in MPS I: a MRI Study ... 538
Outcome of Modified Dega Acetabuloplasty for Congenital Dislocation in Developmental Dysplasia of the Hip Focusing on Internal and External Stabilization ... 538
Orthopedic Myth Hunters: Does Coxitis Fugax forecast a Morbus Perthes?: First Results of a Health Insurance-Data Based Study Design ... 539
Paediatric Hip Sonography at German University Hospitals: The Current Status ... 539
Combined Salter-Pemberton Pelvic Osteotomy ... 540
Forces, Growth Plate Areas and Compressive Stresses at the Proximal Femoral End ... 540
Surgical Treatment of a Large Desmoid Tumour of the Supraclavicular Region: a Case Report ... 540
Esthetic and Functional Improvement in PFFD (proximal femoral focal deficiency) with Deficient Hip Joint Development (Pappas type II–III) by Rotation Plasty Winkelmann Type BIII ... 541
1 Year Follow up After AMIC Therapy in Osteochondral Necrosis of the Femoral Head in an 17 Year Old Football Player ... 541
Progressive Valgus Deformity of Distal Femur Following Supracondylar Osteotomy: Report of 3 Cases ... 541
Successful Management of Acute Dislocation of the Hip in Down syndrome: A Case Report and Review of Literature ... 541
Modern Strategies in the Treatment of Proximal Femoral Focal Deficiency (PFFD) of Different Severity: Primary Prosthetic and Orthotic Treatment in Respect of the Pappas Classification—Chances and Limits ... 542
Diagnostic and Treatment Concept of Femoral and Tibial Torsion Disorders at the OKS ... 542
Lengthening of Long Bone with Unilateral Fixator over Plate ... 543
Operative Treatment of Severe Skeletal Malformations of the Lower Extremity Caused by Phosphate Diabetes ... 543
Hamstring Lengthening for Therapy of Crouch Gait Might Lead to Muscle Weakness and Stiff Knee Gait in Patients with Cerebral Palsy ... 543
Serial Casting and Redressement Therapy in Neuromuscular Diseases ... 544
Molding Helmets: Experiences of >1000 Cases ... 544
Trampoline Related Injuries in Children. A Summary of 65 Cases ... 544
Subfascial Closed-Suction Drains do not Reduce the Incidence of Surgical Site Infections in Musculoskeletal Hip Surgeries in Children ... 545
Ulnar Longitudinal Deficiency in Patients with Hereditary Multiple Exostosis ... 545
Percutaneous Release of Trigger Thumb in Children: Surgical Technique, Review of the Literature and Presentation of 6 Own Cases ... 545

## Undesirable Treatment Results

#### *Undesirable treatment results V1.01*

### *Posttraumatic Stiff Elbow in Children: What Outcomes Should be Expected After Surgical Treatment?*

Cohen, E.*[1], Alkian, T.[1], Atar, D.[1, 2]

[1]Soroka Medical Center, Ben Gurion University, Orthopaedics, Beer Sheva, Israel, [2]Soroka Medical Center, Orthopaedics, Beer Sheva, Israel

***Question*** A stiff elbow is usually defined as having movement <30° in extension or <130° of flexion. The critical range of motion (ROM) is 30°–90°. The prevalence of elbow stiffness as a complication of trauma in the pediatric population is not known. We observed unpredictable results in our patients.
***Objective*** The study hypothesis was that results of surgery for post traumatic elbow stifness in children are unfavorable. We intended to check the hypothesis and see in which circumstances the outcomes are good or not.
***Methodology*** The study is a retrospective study series. The time frame was 2002–2011 and based on the cases of an university hospital. Based on ICD-9 codes, we found 900 children aged 2–16 years who were operated for either a medial epicondyle/lateral condyle or supracondylar humeral fracture.
Eight children suffered from stiff elbow; we were able to follow six of them. Six underwent surgery (open arthrolysis—5 patients, arthrodiastasis using Ilizarov technique—1 case). Physiotherapy was part of the protocol. Evaluation is based on the last follow-up examination, which included: ROM, neurological status, and pain. Radiographs were taken. The mean follow up was 1 year and 8 months (range 0.4–4.2 years).
We arbitrarily defined a good result if the patient had at least 90° arch of motion and was free of pain; fair result if the arch of motion was at least 60° degrees and the patient had no pain; poor result if the patient had either reduced ROM or pain. Among the patient cohort there was a good result, two fair results and three poor results—one after arthrodiastasis and two after open arthrolysis.
***Result*** Posttraumatic stiff elbow in children is rare. Children more than 10 years old and children who underwent primary surgery after at least 1 week appear to have a reduced risk for stiff elbow. The risk is lowest after supracondylar fracture and considerable after medial epicondyle fractures (3 of 52 patients). 30° of longtime improvement in the range of motion is a real expectation but results of surgery are unpredictable. Mayo Clinic Performance Score is popular for evaluation of elbow function in adults but is not applicable in pediatric population. There is any other accepted elbow assessment score suitable for children. We found no similar studies, to compare our results with other centers.

#### *Undesirable treatment results V1.02*

### *Recurrence of Axial Malalignment After Removal of Temporary Hemiepiphysiodesis*

Farr, S.*[1], Radler, C.[1], Meizer, E.[2], Ganger, R.[1]

[1]Orthopädisches Spital Wien Speising, Abt. für Kinderorthopädie und Fusschirurgie, Wien, Österreich, [2]Orthopädisches Spital Wien Speising, I. Orthopädische Abteilung, Wien, Österreich

***Question*** Temporary hemiepiphysiodesis using a plate and screw system has become a standard procedure to correct frontal malalignment of the lower extremities in the growing child. In most cases removal of the plates is performed before skeletal maturity with subsequent growth and the possibility of rebound and recurrence.

***Objective*** We analyzed consecutive patients after removal of temporary hemiepiphysiodesis to evaluate recurrence and possible influencing factors.

***Methodology*** We included patients from a prospective consecutive database who had a correction of an idiopathic valgus or varus deformity by temporary hemiepiphysiodesis at the distal femoral and/or proximal tibial growth plate using the eight-plate. Only patients who had all implants removed for at least 1 year and had reached skeletal maturity were included. The study included a clinical examination and a long standing radiograph of both lower extremities in bipedal stance. The change of the mechanical axis from implant removal to the time of follow-up at skeletal maturity was evaluated and a statistical analysis was performed regarding the influence of age and body mass index (BMI) at removal.

***Result*** Twenty-nine patients presented for examination. Three patients had temporary hemiepiphysiodesis a second time due to recurrence after the first correction and were included as separate cases which resulted in 64 extremities for evaluation. All patients had bilateral correction with 58 extremities presenting valgus and 6 varus malalignment. Hemiepiphysiodesis was performed on the femur in 37 extremities, the tibia in 17 extremities and on both in 10 extremities. The mean age of the 14 female and 15 male patients was 13 years at the time of implant removal and 17 at the time of evaluation. A recurrence in the direction of the original malalignment was seen in 27 extremities (42 %) with the mechanical axis changing a mean 16 mm (range 5–48 mm). A change of the mechanical axis away from the original malalignment was observed in 17 extremities (27 %) with the mechanical axis changing a mean 9 mm (range 4–17 mm). No change defined as change of 3 mm or less occurred in 20 extremities (31 %). Estimating a possible growth until age 14 for girls and age 16 for boys the group with a change of 3 mm or less had a mean 0.8 years of growth remaining versus 2.3 years growth remaining in the recurrence group. However, using a spearman's correlation neither age nor BMI at removal had a statistical correlation with the amount of change of the mechanical axis.

#### *Undesirable treatment results V1.03*

### *Spastic Gastrocnemius Fascicles Do Not Lengthen When Braced with AFOs*

Hösl, M.*, Böhm, H., Döderlein, L

Behandlungszentrum Aschau GmbH, Orthopädische Kinderklinik, Aschau im Chiemgau, Deutschland

***Question*** In children with cerebral palsy (CP), orthotic bracing of the ankle is a favourable treatment in non-fixed spastic equinus deformities. It relies on low load tensile stress applied over several weeks to lengthen the muscle–tendon unit (MTU). While prolonged stretch is a likely stimulus for muscle growth, immobilization may yet promote atrophy.

***Objective*** To assess the effects of AFO bracing on spastic gastrocnemius muscle morphology.

***Methodology*** Fifteen children's, aged 10 ± 3 years, with uni- or bilateral spastic CP, GMFCS I or II and equinus participated. 13 typically developing (TD) children (age: 9 ± 2 years) served as controls. CP children were analyzed before and after 15 ± 8 weeks of AFO treatment, 5 with full-time, 10 with night bracing. Gastrocnemius medialis (GM) fascicle length (FL), length of the distal GM tendon (TL) and whole MTU length were captured by standard ultrasonography in combination with 3D motion tracking (Vicon) during passive movements. Participants were seated, the knee maximally extended and their ankle was manually moved 3–5 times within the available ROM. All parameters were normalized to shank length. First, cross-sectional comparison between CP and TD were made. Secondly, pre- and post AFO data were analyzed.

***Result*** Prior to bracing, max length of the entire MTU during passive stretch was 118.0 ± 1.1 % of shank length in TD, while it was significantly shorter in children with CP (113.8 ± 3.3 %, $p < .001$). Mean max FL tended to be shorter in CP (9.8 ± 2.7 vs. 11.3 ± 1.5 %, $p = .086$) and, overall, fascicles also were less extensible ($p = .081$). The TL strain indicated less extensibility of the tendon, too (11.7 ± 3.8 vs. 16.7 ± 6.9 %, $p = .022$). Max distal TL was not different 51.0 ± 6.1 vs. 50.1 ± 5.4 %. Therefore much of the MTU difference was caused by shorter spastic muscle bellies in CP.

After bracing, max MTU and TL were nearly unaltered. Besides, no differences concerning their extensibility could be noted. However, max FL was significantly further reduced to 8.1 ± 1.7 % ($p = .013$) and fascicles also tended to be less extensible. At comparable muscle length, shorter fascicles may reflect decreased muscle volume after bracing. Our results likely display the consequences of fascicle atrophy. Sarcomeres may have been lost instead of being added. Much of the tensile stress of AFO bracing in spastic equinus may affect the soleus only. In conclusion, to induce lengthening of the bi-articular gastrocnemius, it seems necessary that orthotics need to immobilize the knee in extension, too.

#### *Undesirable treatment results V1.04*

### *Growth Disturbances of the Distal Tibia After Circumferential Release for Idiopathic Clubfeet: 10 Years Follow-up*

Stücker, R.*[1], Tettenborn, L.[2], Burghardt, R.[1]

[1]Altonaer Kinderkrankenhaus, Kinderorthopädie, Hamburg, Deutschland, [2]Universität Hamburg, Deutschland

***Question*** There is some evidence in the literature that distal tibial deformities may occur after clubfoot surgery but the true incidence is yet unknown.

***Objective*** The aim of this study was therefore to evaluate the incidence of deformities of the distal tibia in patients with idiopathic clubfeet underwent surgical intervention with a circumferential release in early infancy.

***Methodology*** A retrospective follow up evaluation of children with idiopathic clubfoot was conducted. We reviewed children who had undergone surgical treatment by a circumferential release in our department after unsuccessful casting, and who were at least 10 years of age. Main focus was the measurement of anterior posterior and lateral view radiographs for distal tibia deformities. Of 65 patients (93 feet) who had surgery for idiopathic clubfoot from 1998 to 2002 radiographic data of 35 patients (52 feet) were analyzed. There were 21 boys and 14 girls, 18 had unilateral and 17 bilateral clubfeet. Mean age at follow-up was 11 + 5 years (range 10 + 5–13 + 3 years).

***Result*** An anteflexion deformity was present in 25 of 52 feet (48.1 %) and a valgus deformity in 29 of 52 feet (55.8 %). 18 feet (34.6 %) had a valgus deformity <84° requiring surgery by epiphyseodesis. There was no correlation between hindfoot valgus and radiographic ankle valgus deformity. There was also no correlation between decreased ankle dorsiflexion andradiographic anteflexion deformity of the distal tibia. A flat-top-talus was evident to some degree in all cases. This is the first study, to our knowledge, highlighting the high incidence of distal tibial deformities after surgery for idiopathic clubfoot. It is unclear at this time if this is also true for clubfeet after conservative treatment. However, patients with clubfeet should have radiographs of the ankle joint between the ages of 10–12 years in order to be able to treat possible deformities through guided growth.

### Torsionsmalalignement

***Torsionsmalalignement V2.01***

#### *Classification of Rotational Deformities of the Legs in Children and Adolescents and Their Associated Gait Patterns*

Multerer, C.*, Böhm, H., Hösl, M., Döderlein, L

Orthop. Kinderklinik Aschau, Deutschland

***Question*** Congenital rotational deformities of the lower limbs might be associated with a variety of clinical problems: children are typically referred to the outpatient clinic because of their noticeable in- or out-toeing gait, often associated with worn out footwear. Adolescents frequently complain about knee and hip pain.
***Objective*** It is well known that the foot progression angle can be normal even in presence of torsional deformity. The aim of this prospective study is to define classes of rotational deformities and show their associated gait deviations.
***Methodology*** Fifty-eight patients with rotational malalignment of the femur and/or tibia, older than 8 years were recruited. A rotational profile of tibial torsion and femoral anteversion was determined with MRI or CT scans. All patients were clinically examined and underwent a 3D gait analysis.
Alignment of the femur and tibia might involve nine possible classes. E.g. the first class (IAIT) is defined as increased anteversion and increased tibial torsion; class IANT involves increased anteversion with normal tibial torsion and so on. NANT is the control group without any malalignment. For each group foot progression angle (long axis of the foot relative to the direction of walking), hip and knee rotations and knee abduction moment were reported. Correlations between MRI, clinical rotations and rotations during gait were calculated.
***Result*** Most patients showed normal or increased anteversion with all combinations of tibial torsion, decreased anteversion was less common. For all classes the sum of hip and knee rotation during walking corresponds to the foot progression angle. The correlation between MRI and rotations during gait were good for the tibia but low for the femur. This might indicate compensatory movements at the hip during walking. Compensatory movements are particularly seen for class NAIT. Although this group has normal anteversion, similar hip internal rotation was observed as in class IAIT compared to controls. This indicates that patients with normal anteversion and increased tibial torsion use compensatory hip internal rotation to decrease their excessive external foot progression angle.
Regarding knee problems, largest twist between femur and tibia was seen for groups NAIT and IAIT that might cause maltracking of the patella and knee pain. In addition foot progression angle might have an effect on knee abduction moment during walking. Therefore pathological load was seen for patients with increased out-toeing DANT and DAIT.

***Torsionsmalalignement V2.02***

#### *Causes of Intoeing: Gait Mechanics in Patients with Reduced Tibial Torsion Versus Patients with Increased Femoral Torsion*

Wegener, R.*[1], Fialkowske, L.[2], Payne, E.[3], Zdenek, K.[3], Klima, H.[3]

[1]Ostschweizer Kinderspital, Abteilung Kinderorthopädie, Labor für Bewegungsanalyse, St. Gallen, Schweiz, [2]Technische Universität Chemnitz, Institut für Angewandte Bewegungswissenschaften, Chemnitz, Deutschland, [3]Ostschweizer Kinderspital, Abteilung Kinderorthopädie, St. Gallen, Schweiz

***Question*** Primary and secondary gait deviations cannot always be clearly distinguished in patients with intoeing gait because they may present with reduced tibial torsion (RTT) or with increased femoral torsion (IFT).
***Objective*** The aim of this study was to compare gait deviations between patients with intoeing gait caused by different torsional deformities.
***Methodology*** Three-dimensional gait data of 18 patients (mean 12.4 years; 25 legs) with RTT (17.6°) and 15 patients (13.3 years; 21 legs) with IFT (38.5°) diagnosed by CT were analysed retrospectively (3 gait cycles/patient). Exclusion criteria were age <10 and >18 years, leg length discrepancy >1 cm, foot deformities, and neurological diseases. Principal component (PC) analysis and linear mixed models were used to compare kinematics and kinetics between both patient groups and a matched, healthy control group (CON). The external moments are reported.
***Result*** Througout the entire gait cycle intoeing (foot progression angle PC1: 77.5 %; $p < .001$) was significantly greater in patients with RTT compared to patients with IFT. While ankle rotation did not differ between the RTT and the CON group ($p = .187$), patients with IFT walked with a significantly greater external ankle rotation compared to the RTT (−1.4° vs. 8.2°; $p < .001$) and the CON group (−1.4° vs. 5.1°; $p < .001$). Internal hip rotation was significantly greater in the IFT group compared to the RTT ($p = .003$) and CON group ($p < .001$; group effect PC1: $p = .027$). The knee adduction moment increased significantly later during the loading response in the RTT ($p = .013$) and the IFT group ($p < .001$) compared to the CON group. Moreover, both patient groups had higher knee adduction moments during midstance and significantly lower knee adduction moments at the end of terminal stance compared to the CON group (PC3).
Increased intoeing is caused by increased hip rotation in the IFT group (secondary deviation) and reduced tibial torsion in the RTT group (primary deviation). The greater prevalence of intoeing in patients with RTT than in patients with IFT has been reported which may be attributed to gait compensation by more externally rotated ankles in patients with IFT (secondary deviation). Greater knee adduction moments may be associated with premature initiation and rate of progression of knee osteoarthritis. In general, differentiating between primary and secondary gait deviations is important for surgical planning and treatment of torsional deformities.

***Torsionsmalalignement V2.03***

#### *Does Rotational Malalignment Cause Knee Pain? Comparison of Gait Between Symptomatic and Asymptomatic Children and Adolescents with Rotational Problems of the Legs*

Böhm, H.*, Multerer, C., Lewens, D., Hösl, M., Döderlein, L.
Orthopädische Kinderklinik, Kinderorthopädie, Aschau i. Chiemgau, Deutschland

***Question*** Idiopathic in- or out-toeing gait may be caused by rotational bony deformities of the leg, e.g. by internal and/or external torsion of the tibia and/or femur. First, it may increase shoe wear and raise individual physical discomfort and cosmetic concerns. While during maturation reports about symptoms are sparse, in adulthood, knee- and hip-arthritis were reported. Biomechanically, in particular increased transverse shift between the thigh and shank during gait might cause abnormal high patellofemoral loads. Yet, rotational deformities as risk factor of knee pain have widely been ignored.

***Objective*** We hypothesized that among children with rotational deformities, in particular those with greater shifts in knee rotation, pain is a typical finding.

***Methodology*** Fifty-eight patients with rotational malalignment of the femur and/or tibia, older than 8 years were recruited. Exclusion criteria were neuromuscular disorders, previous surgeries, cartilage defects, knee varus/valgus, hip- or foot-deformities. Tibial torsion and femoral anteversion was determined with CT scans. In addition pain was assessed and 3D gait analysis was performed. 20 typically developed children served as controls. Mean rotational alignment of the knee during the stance phase of gait was determined by transverse plane rotations of the femur and tibia segment. Patients with knee pain were opposed to patients without knee pain and controls.

***Result*** 14 of 58 patients reported knee pain after longer walking or running. Their mean age was 13.8 [11–17] years, most of them were female (11/14). Compared to normative literature values, bony imaging profiles revealed that 8/14 had normal anteversion and increased external tibial torsion, 6/14 had increased femoral anteversion only. Gait analysis showed that knee rotational alignment in stance was 32° SD = 17° for patients with knee pain, while 16° SD = 20° and 15° SD = 11° for those without pain and controls. Hence, a significant increase of knee torsion could be exclusively noted in patients with knee pain ($p < 0.001$). The prevalence of knee pain was 24 %, involving mainly females that mostly showed external torsion of the tibia, compensatory kneeing in and normal or increased femoral anteversion. During walking torsion of the knee during gait was increased exclusively in patients with pain. Therefore rotational malalignment must be recognized as probable cause for patella overloading syndrome. This gait related parameter might therefore be a potential indicator of derotation osteotomies.

### Paediatric Orthopaedic Problems in the Developing World

***Paediatric orthopaedic problems in the developing world V3.01***

#### *Treatment of the Neglected Clubfoot in the Third World, a Follow up of 6 Years*

Correll, J.K.*[1], Correll, J.[2]

[1]Klinikum Traunstein Südostbayern AG, Unfallchirurgie, Traunstein, Deutschland, [2]Orthopädie am Marienplatz, Kinderorthopädie, München, Deutschland

***Question*** The neglected clubfoot is a common problem in developing countries. There is a high birthrate with a large number of clubfeet, but most patients don't have access to appropriate care. Severe deformities patients are walking on for years often occur. In many cases the forefoot is even facing backwards, due to fixed cavus deformity. Surgical options are limited by lack of aftercare. Full correction must be achieved and maintained during the first and single step of surgery.

In literature only a few articles are focusing on the neglected clubfoot deformity.

***Objective*** The purpose of this study is to show the 6 years results of the operative treatment of neglected clubfeet in the Al Thauwra Hospital in Taiz, Yemen.

***Methodology*** We review our experience of operative treatment of rigid neglected clubfoot deformities in 123 feet, with a follow up of 43 feet. The mean age was 13 years, the mean follow up 20 months. 16 feet were operated on before without any correction. All patients, regardless of their age, were treated with the Ponseti method for 6 weeks preoperatively.

To achieve full correction large bone wedges based on the apex of the anatomy were resected. In 19 cases talectomy was necessary. Bony equinus deformity occurred in all cases and was corrected with a modified Lambrinudi osteotomy. An additional posterior release with Achilles tendon lengthening or in severe cases achillotenotomy was performed. The osseous correction was fixed with K-wires. Postoperatively no weightbearing was allowed for 6 weeks with an above-knee cast, then the K-wires were removed, and patients started walking with a below-knee cast for 6 weeks.

Photo documentation and clinical evaluation were done pre- and postoperatively. The ability to walk, wearing normal shoes and pain were documented, abduction, dorsiflexion and foot load were clinically examined.

***Result*** The clinical results showed sufficient functional correction, all patients were able to walk painless on the sole of their feet in normal shoes. In 4 cases severe equinus deformity returned after 2 years and surgical correction was necessary. In 5 cases overcorrection of abduction occurred with hindfoot valgus deformity, 7 cases had mild cavus deformity left without functional loss. Delayed wound healing occurred in 2 feet. The predicted shortening of the foot was accepted by all patients. The preoperatively performed modified Ponseti method allowed for an easier operative correction of cavus, supinatus and adductus deformity.

***Paediatric orthopaedic problems in the developing world V3.02***

#### *Pediatric Orthopedic Work in Resource Deprived Countries. Transfer of Knowledge by Active Tuition in Tanzania. Example: Blount's Disease*

Exner, G.U.*[1], Ickler, P.[2], Mwakalukwa, L.[2], Hempel, J.[3]

[1]Orthopädie Zentrum Zürich, Zürich, Schweiz, [2]CCBRT, Orthopaedics, Dar es Salaam, Tansania, Vereinigte Republik, [3]Klinikum Straubing, Deutschland

***Question*** During the last 10 years a paediatric orthopaedic service was developed in Tanzania. The interest of the presenter has been to expand his expertise in orthopaedic problems rarely encountered in his University Environment in Europe besides transfer of knowledge to improve orthopaedic service for children in resource deprived (formerly called developmental) countries.

We wish to encourage colleagues to participate in such work and share with them our experience to avoid failures. All authors are actively doing surgery while adhering to Kaye Wilkins' advice ***leave knowledge not scars'.

***Objective*** Example Blount's disease: Langenskiöld has taught us most about diagnosis and management of Blount's disease. Therefore we choose this problem as an example of our activities to introduce adaptation of treatment to means locally available while pursuing the goal of full correction.

Blount's disease is extremely frequent in central Africa and usually only treated when severe deformity has developed. The causes are unknown, but the presenter is convinced that nutritional deficiencies including pre-clinical rickets (also extremely frequent in Africa) are likely to play a major role.

***Methodology*** Standard surgical treatment is high tibial acute osteotomy (HTO) and retention in plaster of Paris.

This does not correct the deformity of the tibial plateau, which to our opinion should be attempted especially in young children with potential for remodelling of the joint surfaces.

***Result*** New Treatment: In the presenters European setup elevation of the medial tibial plateau is performed by an osteotomy guided into the tibial eminence and plate fixation with or without bone graft with simultaneous epiphysiodes of the lateral tibial hemiphysis and the proximal fibular physis. In Tanzania we also use this osteotomy, but fixation is performed with Schanz screw and correction to achieve a neutral Mikulicz line is gradually achieved by external fixation.

***Paediatric orthopaedic problems in the developing world V3.03***

### *Imminent Loss of Extremity Due to Chronic Osteomyelitis and Soft Tissue Infection in Children*

Schnuck, B.*[1], Schulte, M.[2], Brückner, U.[1]

[1]Agaplesion Diakonieklinikum Rotenburg, Kinderorthopädie, Rotenburg/Wümme, Deutschland, [2]Agaplesion Diakonieklinikum Rotenburg, Unfallchirurgie und Orthopädie, Rotenburg/Wümme, Deutschland

***Question*** Our hospital is participating at the Friedensdorf Initiative for several years. Children in war zones worldwide suffering from severe injuries or infections are pre-elected at home to get proper medical treatment in Germany. At admission, extremity loss was imminent in any patient.
***Objective*** The purpose of our study was to evaluate our treatment modalities and results. We point out problems that arose with the special circumstances and with management of severe tissue defects complicated by challenging concomitant diagnoses.
***Methodology*** A total of 18 patients from Afghanistan, Angola and Iraq (13 female, 5 male) were admitted to our hospital. Age ranged from 4 to 13 years. The lower extremity was involved in 17 cases, the upper extremity in 1 case. In 5 cases children presented with bilateral involvement. Main diagnoses were chronic osteomyelitis (n = 12), pseudarthrosis (n = 7), 3° burns (n = 3), amputation injury (n = 1), Buruli ulcer (n = 1) and leprosy (n = 1). Additionally, in several cases general condition was impaired by cachexy, sepsis, sickle cell anemia and worm affection at admission. MRSA and ESBL were evident in 12 cases.
Multiple surgical procedures were necessary for every child in frequent intervals. Surgical treatment included bone resection, arthrodesis, external and internal fixation of bone, multiple wound debridements, tissue reconstruction by free and local flaps, skin transplantation and transplantation of toes. Vacuum assisted closure therapy was used.
***Result*** Mean duration of in-patient treatment was from 1 to 15 months with a total of 25 hospital stays. Preservation of the involved extremity was successful in 94 % of cases. Fracture healing was achieved in all cases. All infections were treated successfully. With return in their home countries all patients were able to ambulate with full weightbearing.
Treatment of complex extremity injuries in children caused by high-energy trauma is very challenging. Deformity, marked soft tissue and bone loss as well as superinfection by multi-resistant organisms were concomitant problems. An interdisciplinary approach is necessary to facilitate proper treatment resulting in restitutio ad integrum. A high consumption of resources must be considered for these patients as well as logistical difficulties when prolonged isolation was necessary. One must be aware of social deprivation due to the complex und long-lasting medical treatment, the absence of attachment figures, cultural differences, foreign language and isolation.

## Hip/Upper Extremity/Free Topics

***Hip/upper extremity/free topics V4.01***

### *Combined Pelvic and Femoral Osteotomy in Patients with Perthes' disease: Clinical and Radiological Outcome at Skeletal maturity*

Rupprecht, M.*[1, 2], Mosow, N.[1, 2], Ridderbusch, K.[1, 2], Stücker, R.[1, 2]

[1]Department of Orthopaedic Surgery, University Clinic Hamburg-Eppendorf, Hamburg, Deutschland, [2]Altonaer Kinderkrankenhaus, Kinderorthopädie, Hamburg, Deutschland

***Question*** Many surgical approaches exist to treat Perthes disease, but there are only few reports in the literature on results of combined pelvic and femoral osteotomies (femoral varus osteotomy combined with Salter innominate osteotomy).
***Objective*** Aim of the present study was to describe the clinical and radiological outcome after combined osteotomies in patients with Perthes disease at skeletal maturity.
***Methodology*** From 1998 to 2009 67 patients with Perthes disease (22 female/45 male; 69 hips) were treated with combined osteotomies (CO) at our institution. Mean age at operation was 7.9 ± 2.3 years. At the time of surgery, 57 % of hips were Catterall group IV, 28 % group III and 30 % group II; in the Herring classification, 67 % hips were group B, 33 % group C. The patients were evaluated clinically and radiologically at skeletal maturity using the Harris-Hip-Score (HHS), the Stulberg classification, and the sphericity index.
***Result*** The average follow-up (FU) was 114 months. Mean age at time of FU was 17.6 years. Based on the Stulberg classification, 48 % were in class I or II, 26 % in class III, and 26 % in class IV or V. The average HHS was 90.2 ± 13.3. Three patients had hip replacement surgery at time of FU. For the Salter osteotomy alone, satisfactory results (Stulberg class I–III) are reported in 78 % [1], respectively for femoral varus osteotomy alone in 76 % [2]. According to these results reported here, the combined osteotomies are safe and effective procedures to treat children with Perthes disease.

***V4.01-2***

### *Quality of Life After Intense Surgery in Patients with Perthes-Disease*

Westhoff B., Palmen N., Rosenthal D., Zilkens C., Krauspe R

Legg-Calvé-Perthes disease (LCPD) is a complex pediatric hip disorder that has a considerable influence on the daily life of children. Different factors can restrict the children in their leisure time activities and may influence their mood. Until now this aspect of the disease has been neglected. Therefore we compared the health related quality of life (HRQoL) of children with severe LCPD who had extensive surgery with pelvic/femoral osteotomy with an age- and gender-matched normal control group.

***Methods*** The KIDSCREEN-10-questionnaire and the modified Harris-hip-score (mHHS) were administered to 17 LCPD-children (16 boys and 1 girl) aged 5–11 years at the time of surgery. Analyses were made retrospectively for the preoperative period and at the time of the follow-up examination at least 2 years postoperatively. The follow-up results were compared to an age- and gender-matched normal control group. Correlations were computed between KIDSCREEN-10 and mHHS pre- and post-operatively.
***Results*** The KIDSCREEN-10-T-value increased from 45.3 (SD 4.6) preoperatively to 70.2 (SD 12.7) postoperatively and was higher than the mean T-value of the control group (57.2 (SD 11.1)). As well the mHHS improved from 54.4 (SD 19.9) to a score of 99.5 (SD 1.5) postoperatively. A strong correlation was found between the preoperative mHHS and the postoperative KIDSCREEN-10-T-value (Spearman's-rho 0.67, $p = 0.003$).
***Conclusions*** Patients with severe LCPD feel a marked reduction in their quality of life during the active stage of the disease—independent of the clinical status of their hip joint. After containment improving surgery and a mean follow-up period of 4.2 years. HRQoL is even better compared with a healthy age- and gender-matched control group. As well an excellent clinical function could be achieved.

***Hip/upper extremity/free topics V4.02***

### *Growth and Deformity after In Situ Fixation of Slipped Capital Femoral Epiphysis*

Druschel, C.*[1], Placzek, R.[2], Funk, J.F.[3]

[1]Charite - Universitätsmedizin Berlin, Centrum für Muskuloskeletale Chirurgie, Berlin, Deutschland, [2]University Hospital Bonn, Clinic for Orthopaedics and Trauma Surgery, Department of Pediatric Orthopedic Surgery, Bonn, Deutschland, [3]Centrum für Muskuloskeletale Chirurgie - Charité Universitätsmedizin Berlin, Kinder- und Neuroorthopädie, Berlin, Deutschland

***Question*** For mild to moderate slipped capital femoral epiphysis in situ fixation is the current treatment standard. However, concerning the implant selection as well as the prophylactic stabilization of the non-affected hip controversies still exist.
***Objective*** The aim of this study was to analyze femoral residual growth and femoral deformities after in situ fixation of SCFE either with k-wires or screws.
***Methodology*** We conducted a retrospective analysis of the radiographs of adolescents treated for SCFE in our department between 01/2003 and 02/2011. To evaluate femoral growth the AT distance, CT distance, CCD angle, pin-joint and pin-physis ratio were determined. Femoral deformity was assessed by measuring sphericity of the femoral head. Degenerative changes were evaluated in the final radiographs. Statistical analysis was performed concerning differences between therapeutically and prophylactically treated hips as well as stabilizations with k-wires and screws.
***Result*** A total of 22 patients (f:m = 14:8, mean age girls: $11 \pm 1$ years, boys: $13 \pm 2$ years) with 26 slipped capital femoral epiphyses were analyzed. k-wires were used for fixation in four hips each therapeutically and prophylactically, 22 hips with SCFE and 14 non-affected hips were stabilized with screws. Treatment with screws did not lead to significantly earlier physeal closure than k-wire pinning. Regarding the femoral growth parameters a significant decrease in the articulo-trochanteric distance and CCD angle was detectable in all groups. The pin-joint-ratio revealed an adequate residual growth in 58 % of the therapeutically and in 72 % of the prophylactically treated hips without significant difference between k-wires and screws. The pin-physis ratio demonstrated similar values. Regarding the femoral deformity the SCFE hips resulted in a significantly reduced sphericity, which remained unchanged during follow-up. The prophylactic stabilization did not result in any deterioration of sphericity.
The results of this study imply that further growth of the proximal femur after insertion of a sliding screw for in situ stabilization of mild to moderate slipped capital femoral epiphysis does occur. Furthermore, an increase of deformity during follow-up through screw fixation as compared to pinning was not noticed. Hence, the assumption that screw fixation leads to permanent physeal impairment cannot be confirmed. The consideration of these results may be helpful for implant selection as well as indicating prophylactic surgery for non-affected hips.

***Hip/upper extremity/free topics V4.03***

### *Arthroscopic Treatment of Septic Arthritis of the Hip in Children*

Fernandez, F.*[1], Eberhardt, O.*[1], Wirth, T.[2]

[1]Klinikum Stuttgart Olgahospital, Orthopädische Klinik, Stuttgart, Deutschland, [2]Olgahospital Stuttgart, Stuttgart, Deutschland

***Question*** The irrigation of the hip joint by hip arthrotomy represents the standard treatment for septic arthritis of the hip in childhood and adolescence. Arthroscopic lavages for joints are well established, but have only reached little acceptance in the therapy of septic hip arthritis in children.
***Objective*** The goal of this study is to evaluate the advantages of the minimally invasive arthroscopic lavage for septic hip arthritis with regards to treatment safety and complication frequency. 20 children aged 6.4 years on average (2–14 years) with obvious signs of septic arthritis of the hip proven clinically and by blood tests were arthroscopically treated. In all cases a histological sample and a bacterial swab was taken. In three children there was a stage I according to Stutz and Gächter, in thirteen a stage II and in four patients a stage III. Because of a concomitant femoral neck osteomyelitis in three cases and an osteomyelitis of the os pubis in a single patient PMMA mini chains were locally administered. In ten children a positive bacterial result was given, with staphylococcus aureus found in 7 cases and single cases with meningococci, salmonella and staphylococcus Warneri. All patients received an intravenous antibiotic treatment for 14 days followed by 4 weeks of oral antibiotics.
***Methodology*** 16 out of 20 children were sufficiently treated by one single arthroscopic lavage. In three cases with additional osteomyelitis a secondary procedure was needed for removal of the antibiotic chain. During follow-up after an average of 2.9 years 19 of 20 children demonstrated a free range of hip joint motion and a full sporting activity without any restrictions. One girl with stage III arthritis and a preoperative duration of symptoms of 7 days developed a femoral head necrosis and chondrolysis with hip joint subluxation. The other 19 patients regained an excellent Harris hip score at follow-up, whereas the girl only produced moderate score values.
***Result*** The hip arthroscopy with lavage represents a safe and minimally invasive method in order to successfully treat septic arthritis of the hip and concomitant femoral neck osteomyelitis in children and adolescents. This technique leads to a very low morbidity offering all advantages of arthroscopic procedures. The use of cannulated instruments through well established safe portals makes this arthroscopic lavage procedure a simple and easily adoptable technique.

***Hip/upper extremity/free topics V4.04***

### *Operative Treatment of the Hands and Feet in Apert Syndrome*

Mann, M.*, Hülsemann, W., Winkler, F., Habenicht, R

Katholisches Kinderkrankenhaus Wilhelmstift, Kinderhandchirurgie, Hamburg, Deutschland

***Question*** The Apert syndrome is a rare syndrome with an incidence of 1:90.000 births. It is easy to recognize due to the craniofacial anomalies and the characteristic syndactylies of the hands and feet.

***Objective*** In the hands our aim is to get a 5-finger hand with satisfying functional results and to minimize the total number of operations by bilateral surgery.
In the feet our aim is that the patients can wear normal shoes and walk free of pain. The problem in most cases is the width of the feet caused by polydactylies. Also the prominent heads of the second and third metatarsal may cause pain.
***Methodology*** In the period from 2001 to 2011 we treated 78 Patients with Apert syndrome. We did 280 operations in the hands and 17 operations in the feet.
In the hands we did 225 syndactyly releases and 58 osteotomies. In 110 syndactyly releases we performed a soft tissue distraction with a Cube-Fix prior to the release.
In the feet we did 33 procedures: 20 osteotomies of the metatarsals and 7 resections of bone duplications. In addition we separated the first and second toe in 6 cases.
***Result*** With our approach we were able to reconstruct a 5-finger hand in 76 patients. We achieved good results performing a soft tissue distraction prior to the syndactyly release: less skin grafts were needed and despite of missing arteries no problems with the blood supply occurred. The wound healing and the cosmetic appearance were better than after a conventional release.
All patients after corrections of the feet wear normal shoes, some of them still need orthopedic insoles.

***Hip/upper extremity/free topics V4.05***

### *5 Year Outcome of Ulnar Osteotomy and External Fixation for Radial Head Dislocation*

Rogalski, M.*[1], Deja, M.[1], Burgemeister, K.[1], Zacher, J.[1]

[1]Helios Klinikum Berlin Buch, Zentrum für Orthopädie und Unfallchirurgie, Berlin, Deutschland

***Question*** Different treatment options in cases of chronic radial head dislocations are reported in the literature. We ask for an critical review of a surgical procedure based on external fixation and ulnar osteotomy.
***Objective*** The aim of this study is to present the results of ulnar osteotomy with lengthening, external fixation and open reduction of radial head.
***Methodology*** We reviewed 20 patients with radial head dislocation. 11 of them with missed Monteggia fracture dislocation, 6 with secondary subluxation/dislocation caused by skeletal dysplasia and 3 congenital cases.
The median age at surgery was 7 years [2 years 6 months–15 years 1 month] in the posttraumatic group. 8 were classified as Bado type I, 2 as II and 1 type IV. The mean delay from primary dislocation to surgery was 2 years and 2 month [1–4 years 2 months]. ROM, functional outcome and radiological data were obtained. The median follow up was 5 years [6 months–8 years 1 month]
***Result*** We have seen 1 redislocation, 1 subluxation, 2 pin infections, 1 delayed union and 2 cases of early arthrosis. Late presented cases [>3 years] after primary dislocation have a poor outcome. We didn't perform ligament reconstruction.
External fixation of ulnar osteotomy provides the advantage of 3 dimensional stepwise correction and allows adjustment in case of recurrent subluxation or dislocation. Final treatment results strongly depend on delay after primary dislocation and surgical intervention as well as normal alignment of the radiohumeral joint surfaces, concavity of the radial head and plasticity in younger age.

***Hip/upper extremity/free topics V4.06***

### *Management of the Tibia in Osteogenesis Imperfect Congenita (OIC) with Special Emphasis on the Choice of Implant, Surveillance and Implant related Complications*

Rogalski, M.*[1], Deja, M.[1], Burgemeister, K.[1], Zacher, J.[1], Fliesenberg, K.[1], Arnold, C.[1]

[1]Helios Klinikum Berlin Buch, Zentrum für Orthopädie und Unfallchirurgie, Berlin, Deutschland

***Question*** Telescopic rodding—especially after introduction of the Fassier-Duval system and sliding technique with ESIN are the preferred methods for femoral correction and stabilization in growing OIC-patients. The surgical treatment of tibial deformity due to this weak bone disease is an ongoing challenge and associated with a high number of complications. The question of the preferred tibial implant in children with OIC has not yet been answered.
***Objective*** Critical review and evaluation of our experience of tibial management in OIC with special emphasis to complications and implant surveillance.
***Methodology*** We reviewed the results of different methods of intramedullary fixation devices after tibial fracture treatment and correction osteototomy at in 32 OIC patients over a time period of 20 years. The surgical techniques included intramedullary stabilization with non elongating single rush rods (n = 21), sliding dual ESIN (n = 4), Bailey-Dubow rodding (n = 12) and the Fassier-Duval system (n = 21). We analyzed fracture rate after treatment, nail and disease related complications and survival rate of each rod.
The median age at surgery for the whole population was 5 years [3–14]. The survival rates (median interval between primary surgery and rod revision) were the following: single rush rod: 30 months, sliding dual ESIN: 28 months, Bayley-Dubow-telescopic rod: 30 months and Fassier-Duval system: 38 months. Complication rate, ROM, functional outcome and radiological data were obtained. The median follow up was 10 years [3–20 years].
***Result*** Rush rods, dual ESIN and BD-telescopic nails are equally effective in terms of surveillance and complication rates in growing OIC patients. Up to now there seems to be a tendency for lower complication rates of the Fassier-duval system.
Refracture, recurrent deformity (antecurvation and bending) and nail migration are still remaining problems and limiting the surveillance rate of different intramedullary devices for the tibial bone in OIC-Patients. Therefore we modified some steps of the surgical procedure and recommendations for postoperative management. Nevertheless some disease related problems still remain and demand an individualized management.

***Hip/upper extremity/free topics V4.07***

### *Ultrasonography of Osteochondritis Dissecans of the Knee Joint*

Demuth, T.*[1], Stücker, R.[1], Meenen, N.M.[1], Rupprecht, M.[1]

[1]Altonaer Kinderkrankenhaus, Kinderorthopädie, Hamburg, Deutschland

***Question*** MRI is currently the modality of choice for the detection and staging of osteochondral injuries, but it is expensive and not readily available. Ultrasound is an approved method for the assessment and monitoring of a wide variety of musculoskeletal disorders.

Aim of this study was (I) to answer the question, whether ultrasonography is able to diagnose and monitor the OD, and (II) to compare the results with the MRI-findings.
***Objective*** From January 2010 to October 2013 24 children (15 girls, 9 boys) affected with an OD of the knee joint were examined by ultrasound and MRI at our institution. The ultrasonic results were analyzed with respect to lesion's localization, stage, size, and stability and were compared to the MRI findings.
***Methodology*** Ultrasonic examination is limited in assessing the OD-stage I, but the fragmentation of the subchondral bone and the condition of the overlying articular cartilage could be assessed in all cases. The size of the lesions varied from a few mm to more than 30 mm in diameter. In OD-stages II–IV, the ultrasound correlates with the MRI regarding defect size and dissection of the OD.
***Result*** Ultrasound is an appropriate tool for the diagnosis and monitoring for OD-lesions staged II–IV. It provides an inexpensive and readily available alternative to the MRI-scan.

### Neuroorthopaedics

***Neuroorthopaedics V5.01***

#### *Functional Monitoring of the Upper Extremity in Children with Spastic Unilateral Cerebral Palsy*

Wolf, S.*, Wenger, P., Rettig, O., Klotz, M., Dreher, T

Universitätsklinikum Heidelberg, Klinik für Orthopädie und Unfallchirurgie, Heidelberg, Deutschland

***Question*** Upper extremity function in children with spastic unilateral cerebral palsy is rather little investigated. Typically the movement pattern of the involved side is compared to the dominant side of non-involved children. This comparison neglects the fact that the involved side typically does not perform unilateral tasks. The finding will therefore not describe the functional ability of the child.
***Objective*** The aim of this study was to monitor unilateral and bilateral tasks of daily living with instrumented 3D motion capture and specifically to observe the involved side assisting the task in comparison to the non-dominant side in typically developing children. Differences in pattern and timing may be used as an indicator for the severity of involvement and may describe malfunction more specifically compared to the manual ability classification system (MACS).
***Methodology*** Sixteen children with spastic unilateral cerebral palsy aged 13.2 (SD 2.3) years (9 female) were recruited and classified according to the MACS. 17 typically developing children aged 13.2 (SD 2.8) years (6 female) were recruited for reference. All children were monitored by a 12 camera motion capture system in the following tasks: (1) Drinking, (2) eating with a spoon, (3) decant cups, (4) turning a key, (5) reaching and pushing a box and (6) tipping the index finger alternating between nose and the top of a bottle on the table ("bottle-nose"). All tasks were performed three times except for "bottle-nose" which was rapidly repeated 30 times. Average movement times and joint ranges of motion for shoulder, elbow and forearm pro-/supination were calculated and compared between groups MACS 1, 2 and 3 as well as to the reference group.
***Result*** Movement times increased relevantly with involvement according to MACS in all 6 tasks. With increasing MACS level maximum shoulder flexion and shoulder abduction increased in all tasks except for drinking. Conversely, maximum elbow extension decreased with increasing MACS level. Maximum forearm pronation was increased with increasing MACS level in drinking, eating, decanting, and "bottle-nose".
***Discussion*** 3D motion capture quantifies functional findings in everyday tasks of children with cerebral palsy which otherwise can only be described qualitatively. The results show a good correspondence between clinical classification according to MACS and joint angle ranges of motion in the performed tasks.

***Neuroorthopaedics V5.02***

#### *Brain Plasticity Related to Hand Motor Intensive Training in Youths with Cerebral Palsy*

Lampe, R.*[1], Blumenstein, T.[1], Turova, V.[1], Alves-Pinto, A[1]

[1]TU München, Klinikum 'rechts der Isar', München, Deutschland

***Question*** Impaired hand motor skills occur frequently in cerebral palsy patients, limiting the performance of daily activities. Improvement of hand motor functions is therefore central in the therapy plan of these patients and in the amelioration of their quality of life. Given the neuronal origin of the motor deficits in these patients and the recognized ability of the brain to change, intensive hand motor training is likely to induce neuroplastic changes.
***Objective*** The effectiveness of intensive hand motor training in cerebral palsy patients was assessed by estimating with functional magnetic resonance imaging the potential neuroplastic changes induced by the training.
***Methodology*** Cerebral palsy patients, aged between 9 and 16 years, received intensive motor training through piano lessons and for 1½ years. Another group of patients received no instrument training during the same time period. Imaging of the brain was done before and after piano training for comparison.
***Result*** The heterogeneity that characterizes this clinical population, in terms of the extension of brain damages causing the deficits and in terms of the clinical symptomatology, was also observed in the range of neuronal changes derived from the imaging data before and after the training. Nevertheless, plastic changes could be registered that point to a beneficial effect of the piano training. The enjoyment and engagement expressed by the youths during the training supports further the musical instrument training as a promising method in the motor and personal development of these patients.

***Neuroorthopaedics V5.03***

#### *Distal Femoral Extension Osteotomy in Children with Cerebral Palsy*

Dreher, T.*[1], Hirsch, K.[1], Wolf, S.[1], Krautwurst, B.[1], Müller, S.[1], Klotz, M[1]

[1]Universitätsklinik Heidelberg, Klinik für Orthopädie und Unfallchirurgie, Heidelberg, Deutschland

***Question*** Children with cerebral palsy often present with crouch gait, which was formerly treated with hamstring lengthening with good initial results [1]. However a high incidence of increased anterior pelvic tilt and recurrence of flexed-knee gait were reported in long-term studies [2, 3]. Hence treatment strategies changed and distal femoral extension osteotomy (DFEO) is now commonly used for correction [4, 5]. However there are no studies investigating changes in gait beyond a follow-up period of 1 year after DFEO.
***Objective*** The aim of this study was to investigate the functional outcome following more than 1 year after DFEO. The crucial question was if DFEO leads to an adequate improvement of knee function without a relevant increase in anterior pelvic tilt.

***Methodology*** Twenty-three ambulatory children with bilateral spastic cerebral palsy (GMFCS Level II–III) and crouch gait were included in this study. They all were treated with DFEO in the context of multilevel surgery. According to a standardized protocol the patients underwent clinical examination and three-dimensional gait analysis before and 3 ± 1 years after surgery. A 90° blade plate was used to fix the femoral bone in all osteotomies. T tests were used to calculate pre/post differences and the level of significance was set at 0.05.
***Result*** In all cases the femoral bone healed without problems. Concerning gait function a significant ($p < 0.001$) improvement of knee motion towards extension during stance phase (from 33.4 to 18.2 degrees of flexion) was found. This indicates that DFEO is an effective procedure in the context of multilevel surgery to treat crouch gait. The results for the knee joint are comparable with those of hamstring lengthening [1–3]. However, pelvic tilt deteriorated significantly ($p < 0.05$) from 14.2 to 17.7 degrees after surgery indicating that increase in anterior pelvic tilt also represents a problem when DFEO is used. This may potentially be explained by a reduction of hamstring strength as a result of de-tensioning after slight femoral bone shortening which is commonly done during DFEO procedure. It needs to be further investigated if shortening of the femoral bone relevantly influences hamstring strength in these patients.

***References***
[1] Kay RM et al (2002) J Pediatr Orthop, 22:169–172
[2] DeLuca PA et al (1998) J Pediatr Orthop, 18:712–718
[3] Dreher T et al (2012) J Bone Joint Surg, 94:121–130
[4] Stout JL et al (2008) J Bone Joint Surg, 90:2470–2484
[5] Novacheck T et al (2009) J Bone Joint Surg, 91(2):271–286

### Foot and Lower Leg

***Foot and lower leg V6.01***

#### *Pressure Distribution of Children with Clubfoot Treated with the Ponseti Method and Operative Methods*

Kranzl, A.*[1], Mindler, G.[2], Radler, C.[2]

[1]Orthopädisches Spital Speising, Labor für Gang- und Bewegungsanalyse, Wien, Österreich, [2]Orthopädisches Spital Wien Speising, Abt. für Kinderorthopädie und Fusschirurgie, Wien, Österreich

***Question*** The Ponseti method is a non invasive clubfoot treatment with increasing utilization all over the world. The short-term successes are well documented in the literature, only a few studies report on the mid-term results. The present study used pressure distribution in order to evaluate the midterm results of the Ponseti method and to compare the outcome to surgical clubfoot treatment results.
***Objective*** We compared patients from our prospective database for clubfeet treated with the Ponseti method with a minimum age of 3 years and compared the results with a previous study conducted by us on the subject of surgical clubfoot treatment. Pressure distribution were done on unilateral and bilateral affected patients.
***Methodology*** Thirty-two children with unilateral (13 feet) and bilateral clubfeet (38 feet) were examined at a mean age of 5.9 years (3.1–8.3 year). All feet were initially treated with the Ponseti method including percutaneous Achilles tenotomy and 6 (12 %) feet did require additional minor surgery. Results were compared to two groups of surgical clubfoot treatment (McKay's procedure 32 feet and treated with a soft tissue release called "mini-cincinnati" 40 feet). All results were compared to a reference group (n = 34 feet). For analyzing the pressure distribution we divided the foot in 10 regions. Parameters analyzed: Gait line, peak pressure, contact area and peak force.
***Result*** We found a lateralization of the gait line in all patient groups. The Ponseti group showed a pattern that was closer to the reference pattern. Comparing the peak pressure pattern higher pressure values were found in the medial lateral mid foot region. Lower peak pressure values in the heel region were seen in all clubfoot groups compared to normal. Contact area (whole foot) was always greater in all other groups compared to the reference group. Peak pressure and peak force in the lateral fore and mid foot were always higher in the surgical group compared to the Ponseti group. Pressure under the MT head was reduced in all patient groups but the Ponseti group showed values which were closer to normal.
Pressure distribution gives more objective information in evaluation of the outcome of surgically or conservatively treated clubfeet. Although all patients were satisfied with the results we found significant differences which may indicate functional problems later in adulthood. The Ponseti method showed better outcome results compared to the surgical groups with only minor deviations from normal.

***Foot and lower leg V6.02***

#### *Congenital Vertical Talus: In Search of the Best Treatment Algorithm*

Horter, M.*[1], Vogt, B.[1], Schuhknecht, B.[1], Tretow, H.[1], Schiedel, F.[1], Rödl, R[1]

[1]UKM, Kinderorthopädie, Münster, Deutschland

***Question*** Congenital vertical talus is a rare rigid flatfoot deformity. The treatment of this deformity is necessary to prevent pain and disability. The main treatment techniques are the adapted peritalar release with extensive soft-tissue releases and adapted tendon-transfer and casting and the Dobbs-procedure that consists of serial casting and afterwards minimal surgery (percutaneous tenotomie of the Achilles tendon and talonaviculare pin fixation).
The purpose of the present study was the comparison of the efficacy of both methods.
***Objective*** Since 2004 we treated 14 patients with 19 vertical tali. Before 2008 we preferred the adapted peritalar release (8 cases), since 2008 we followed in 11 cases the protocol of Dobbs. The clinical datas, the classification of Hamanishi and Ogata & Schoenecker, the kind of surgery, and the clinical and radiological results were documented. To evaluate the radiological result we used the Hamanishi score.
***Methodology*** We performed correction radiographically in 18 of the 19 feet. The ankle plantar flexion improved in both groups, not the dorsiflexion. In both groups we had none operation-obligatory complication and no talus necrosis. The average age at operation date was much lower in the Dobbs group: 1.24 versus 3.48 years. According to the Hamanishi classification we have in the Dobbs-group 2 excellent, 3 good results, 4 subluxated feet and 2 luxated feed. In the peritalar-release-group we have 2 excellent, 5 good results, 1 subluxated foot and no luxated foot. The procentual TAMBA-improvement was much higher in the group treated with peritalar release: decreasing of 26.58 % of the preoperative angle versus 48.38 % in the Dobbs-group. The results showed no correlation with the preoperative classification of Hamanishi and Ogata & Schoenecker or the age of the child. We were not able to find a predictable mark, which foot is profiting by which treatment.
***Result*** So far there is no clear treatment algorithm like in clubfoot. In generally both treatment possibilities are successful. In our collective the results of the peritalar release group are much better, the children did not show more complication, but they needed a more invasive treatment. We postulate that an early treatment attempt with the less invasive Dobbs-procedure is recommendable, although of the risk of relapse. In this case a more invasive treatment like the adapted peritalar release should be discussed to increase the results and to prevent compromisation.

***Foot and lower leg V6.03***

### *A Prospective Radiological Study for Treatment of Iuvenile Flexible Flatfoot: Effect of the Calcaneus Stop Screw to the Heel Valgus*

Fingernagel, T.[1]*, Hofstätter, S.[1], Ullmann, D.[1], Trieb, K.[1]

[1]Klinikum Wels-Grieskirchen, Orthopädie und orthopädische Chirurgie, Wels, Österreich

***Question*** The Calcaneus stop screw is a clinical reproducible and effective operation technique to correct the iuvenile flatfoot. This is the first study which evaluates the effect of the calcaneus stop screw on the heel valgus by use of the Saltzmann view beside other parameters. The Saltzmann view reflects the heel valgus between tibia and calcaneus and is a standard X-ray examination of hindfoot pathologies in adults.
***Objective*** The aim of our study was to documented the effect of the calcaneus stop screw on the TMT-I-angel and especially on the heel valgus which is measured with the Saltzmann view angel in a standardized X-ray.
***Methodology*** We treated 28 feet in 18 patients with a symptomatic flatfoot. All patients did not improve with physical treatment for at least 3 months. The mean age at operation was 11.6 years (between 8 and 14 years). We treated 12 boys and 6 girls. All operations were done in a standardized manner by two surgeons with a cannulated screw with a diameter of 4.5 mm. We did not perform any additional soft tissue surgery. In addition to the clinical correction our main focus was to the objective radiological parameters including the TMT-I-angel and especially the Saltzmann-view. Our radiological parameters were done before operation, 3 weeks after and afterwards once a year till to the removal of the screw. All values were statistically analyzed. Postoperative all patients used two crutches for 2 weeks half weight bearing.
***Result*** The mean follow up time was 20.3 months. Two screws were already removed routinely (after 21 and 39 months). In 16 patients (26 feet) a very good improvement of the foot position was achieved and also documented on the podoskop. The TMT-I-angel improved significantly from 16.3° preoperative to 6.2° after 3 weeks and to 5.7° after 1 year ($n = 17$ feet). The heel valgus documented by the Saltzmann view changed significantly from 18.3° preoperative to 6.3° after 3 weeks and to 9.3° after 1 year ($n = 16$ feet). In one case a transient overcorrection was observed which diminished after 1 year. In one case there was a short dysaesthesia which was fully resolved after 2 weeks; the peroneale spasm which is quite often mentioned in literature did not occur in our group. All screws were in unchanged position at follow-up. After 12 months we saw in 5 feet a seam around the screw without any clinical consequence. In one patient a loosening of the screw needed revision.

***Foot and lower leg V6.04***

### *Total Tibial Agenesia: Therapeutic Options and Results of Treatment*

Schuhknecht, B.[1]*, Wacker, S.[1], Horter, M.[1], Schiedel, F.[1], Rödl, R[1]

[1]Universitätsklinikum Münster, Kinderorthopädie, Deformitätenrekonstruktion und Fußchirurgie, Münster, Deutschland

***Question*** Tibial hemimelia is a very rare disease with an incidence of 1 in 1,000,000 live births (Fernandez-Pallazi 1998). It is characterized by deficiency of the tibia with relatively intact fibula.
The most current classification for tibial hemimelia is the Weber Classification. It divides the tibial malformations into seven main groups (Weber 2008).
The most common type is the total tibia agenesia with a single fibula (Type VII) with 62 % of the cases.
In addition to the classification of tibial malformations, Weber uses a score system including the whole leg and all main functions to represent the grade of impairment. The higher the score number the lower is the grade of impairment.
***Objective*** We report the results of treatment for 13 limbs in 10 patients with unilateral or bilateral tibial agenesia classified as type-VII B according to the Weber Classification to discuss the different therapeutical options and clinical outcome.
***Methodology*** The average age at first treatment was 2.4 years. The average current age is 9 years (DOB 1983–2011).
According to the Weber score system, the classes of defect for the affected patient's limb were 4 and 5 with an average score number of 8.4.
Two different types of treatment were applied according to the parents' decisions:
8 patients (10 limbs) received limb salvage procedures (mean age at first treatment 2.13 years).
2 patients (1 unilateral, 1 bilateral) received a disarticulation of the knee, one as the primary procedure (unilateral tibial agenesia aged 1 year), the other one after the attempt of deformity correction (aged 13 years, bilateral disarticulation).
***Result*** The 8 limb salvage treatments included foot centralization and fibular transfer in 8 limbs and callus distraction lengthening in one limb. The average numbers of operations for each patient were 3,625.
None of the 10 knees treated by fibular transfer achieved an orthesis-free functional result, but all patients are currently able to walk in ortheses. For 2 patients additional surgery is planned in near future.
Both patients with disarticulation of the knee are able to walk and run in their prostheses.
Thus even in low score numbers (Weber score class 4 and 5) limb salvage procedures result in good function of the leg, but the amount of time and surgical treatment is clearly longer than in primary knee disarticulation.
Therefore therapeutic options including knee disarticulation should be discussed in detail with the parents to find the best individual treatment for each patient.

***Foot and lower leg V6.05***

### *Long-Time Consequences of Avulsion Fractures of the Proximal Tibia in Adolescence*

Wack, C.*[1], Angáni, L.[1], Wirth, T.[1], Fernandez, F[1]

[1]Klinikum Stuttgart Olgahospital, Orthopädische Klinik, Stuttgart, Deutschland

***Question*** Avulsion fractures of the tibial tubercle are a very rare injury in adolescence, mostly relating to male gender. The classification of these fractures was made and modified according to literature by Ogden. A statement about the occurrence frequency of growth deformities and about their long-time consequences cannot be made up to now.
***Objective*** Aim of this study was to show if there are correlations between type of tibial tubercle fracture, treatment and development of late complications.

***Methodology*** We retrospectively reviewed all tibial tubercle fractures treated in our department from 06/2005 to 12/2013. The classification of these fractures follows the Ogden classification on the basis of a lateral X-ray of the knee. Data included the age at the time of injury, gender, affected side, the trauma mechanism, treatment, comorbidity and complications.
***Result*** Overall there have been treated 31 tibial tubercle avulsion fractures (25 of them were male). In one boy there has been two independent avulsions during the period of investigation, another boy showed bilateral injuries of tibia tubercle. Causes of injuries were jumping in 11 cases, ball sports in 12 cases, during running in 3 cases and a direct trauma in five cases, 60 % were accidents in school. The mean age was 14.4 years (12.7 in girls, 14.8 in boys). In 70 % the left side was affected. Accordingly to the Ogden classification we had 9 type 1-, 13 type 2-, 9 type 3-injuries. In about 68 % an operative reposition was necessary. In one patient we found Osgood Schlatter's disease as a possible predisposing cause.
Five patients had a leg length discrepancy smaller than 1 cm, one patient was in need to therapy with a preterm bridging and one child with a patella baja plus genu varum et recurvatum. In four cases an additional surgery treatment was necessary to revise the deformity.
Because of the anatomy of the adolescence proximal tibia with apophysis and epiphysis, it has a high potential for bone deformity in that area. Fractures through the proximal tibia epiphysis should be set to prevent a bridging, while fractures in the tibia plateau should be set exactly.
The data represent that patients with tibial tubercle fractures have to keep under control until the end of bone growth to realize and set an early treatment to use the last potential of correction.

***Foot and lower leg V6.06***

#### *Accordance of Preoperative Magnetic Resonance Imaging (MRI) and Arthroscopic Findings in Juvenile Osteochondritis Dissecans (OCD)*

Utzschneider, S.*[1], Roßbach, B.P.[1], Arnoldi, A.[2], Heimkes, B.[1], Müller, P.[1], Jansson, V.[1]

[1]Ludwig-Maximilians-Universität, Klinik und Poliklinik für Orthopädie, Physikalische Medizin und Rehabilitation, München, Deutschland, [2]Ludwig-Maximlians-Universität, Radiologie, München, Deutschland

***Question*** In clinical diagnostics of juvenile osteochondritis dissecans (OCD) magnetic resonance imaging (MRI) is widely used to determine the stadium of the OCD according to the classification system by Dipaola et al. It is common that stage I/II cases are treated conservatively based on the MRI findings. Clinical experience shows that several stage I or II patients have persistency of pain despite conservative therapy over 6 months. Analogous to these symptoms, many of these patients undergo secondary diagnostic and therapeutic arthroscopy.
***Objective*** The Hypothesis was that the staging of Dipaola et al. for juvenile OCD in the preoperative MRI does not correspond to stages collected in arthroscopy. Furthermore, the authors investigated if 3 Teslan MRI provides any information gain in juvenile OCD compared to 1.5 Teslan MRI.
***Methodology*** Sityy-three juvenile patients (37 female/26 male; mean age 12.5; 8–16 years) with OCD underwent arthroscopy after MRI imaging in our department. In 54 cases closed MR scanner with a magnetic flux density of 1.5 Tesla, in nine patients closed 3 Tesla MR scanner were used. Staging of OCD was identified with the classification system of Dipaola et al. All arthroscopic procedures were performed by experienced orthopaedic surgeons with specialization in arthroscopic treatment. Arthroscopic findings were compared with those of the preoperative MR imaging (evaluated by an orthopaedic surgeon and an radiologist) in each patient after surgery.
***Result*** Magnetic resonance imaging (MRI)/arthroscopy revealed in accordance to Dipaola OCD stage I in 4/19 patients, stage II in 31/22 patients, stage III in 22/9 patients and stage IV in 6/6 patients. In arthroscopy no pathology was found in seven patients. In total, a correct diagnosis was only made in 26 of 63 cases (41.3 %) by MRI. In particular, stage II and III were assessed too high or too low by MRI in 50 %. Inter alia in two cases of MRI diagnosed stage I patients during arthroscopy stage III was verified. With 3 Tesla MRI a correct diagnosis was also found only in 44.4 %.
The findings in MRI, including the classification system of Dipaola et al., should not be used as single criterion for the decision if conservative or surgical treatment is adequate. 3 Tesla MRI gives no superior information gain in juvenile OCD.
In juvenile patients having an OCD stage I or II according to Dipaola et al. in MRI, who do not show declining symptoms under conservative treatment, a diagnostic arthroscopy should be considered to exclude worse stages of the OCD.

### Spine

***Spine V7.01***

#### *Long-term Follow-up after Posterior Hemivertebra Resection with Transpedicular Instrumentation in Young Children with Congenital Scoliosis*

Ruf, M.*[1], Letko, L.[1], Merk, H.[2]

[1]SRH-Klinikum Karlsbad-Langensteinbach, Spinenchirugie, Karlsbad, Deutschland, [2]Ernst-Moritz-Arndt Universität Greifswald, Klinik für Orthopädie und Orthopädische Chirurgie, Greifswald, Deutschland

***Question*** Posterior hemivertebra resection with transpedicular instrumentation is a standard procedure in the treatment of congenital scoliosis. Early correction to avoid secondary compensatory curves is recommended. However, there is a lack of tong-term results referring to further spinal growth.
***Objective*** To evaluate the long-term effect of surgery in very young children the following study was conducted.
***Methodology*** From 6/1991 to 2/2003, 24 children aged 1–4 years (mean 2 years 8 months) were operated on by posterior hemivertebra resection with transpedicular instrumentation. Twenty-two patients (28 hemivertebrae) could be included with a minimum follow-up of 10 years (mean 13 years 1 month). Twelve patients had single hemivertebrae, 10 patients had complex deformities with hemivertebrae, bar formations, and rib synostosis. Sixteen hemivertebrae were located in the thoracic spine, 8 thoracolumbar and 4 in the lumbar spine.
***Result*** Average 2.7 (1–9) segments were fused. Cobb angle of the main curve averaged 51.2° (16–109°) preoperatively, 16.1° (−2 to 45°) postoperatively, and 15.2° (−4 to 81°) at latest follow-up. The secondary cranial curve improved spontaneously from average 18.1° (2–50°) preoperatively to 5.7° (−2 to 17°) postoperatively and 3.3° (−8 to 24°) at latest follow-up, the secondary caudal curve respectively from 22.9° (5–89°) to 8.0° (−8 to 34°) and 8.4° (−14 to 95°). In the subgroup of patients with single hemivertebrae the main curve measured 35.1° preoperatively, 9.3° postoperatively, and 7.2° at follow-up, in the subgroup with complex deformities respectively 68.7°, 23.6°, and 24.0°.

Complications and further operations: 4 implant failures (revision surgery), 1 pedicle fracture (revision), 1 dural tear (suture), 1 infection (implant removal/new instrumentation), 9 surgeries following new deformities, 9 implant removals.
There were no neurologic deficits and no narrowing of the spinal canal during further spinal growth.
Radiographic follow-up demonstrated bone growth despite the instrumentation with a bony narrowing at the waistline of the fusion area similar to a bloc vertebra.
***Discussion*** Posterior hemivertebra resection with transpedicular instrumentation is a safe and efficient procedure to correct congenital scoliosis early and to control the deformity until the end of growth. Long fusion segments and the development of secondary structural curves can be avoided. Regular follow-up is necessary to intervene again in case of new developing deformity.

***Spine V7.02***

### *Results of 10 Years with the Growing Rod System in the Treatment of Scoliosis*

Wimmer, C.*[1, 2, 3], Pfandlsteiner, T.[4]

[1]PMU Salzburg, Deutschland, [2]Klinik für Spinenchirurgie Vogtareuth mit Skoliosezentrum, Schön klinik Vogtareuth, Deutschland, [3]Klinik für Spinenchirurgie Vogtareuth mit Skoliosezentrum, Kirchanschöring, Deutschland, [4]Klinik für Spinenchirurgie Vogtareuth mit Skoliosezentrum, Deutschland

***Question*** Seit 2002 wurden wachstumslenkende operative Verfahren mit Pedikelschrauben in Kombination mit mitwachsenden Stäben verwendet. Von 2002 bis 2013 wurden 73 Patienten mit Pedikelschrauben basierten wachstumslenkenden System operiert. Die Diagnose war kongenital-, neuropathische-, oder eine infantile Skoliose.
Studiendesign: Prospektive, offene, unkontrollierte monozentrische Studie zur Überprüfung der wachstumslenkenden Instrumentation.
***Objective*** Die Komplikationsrate und die Korrektur im Langzeitverlauf nach zu untersuchen
***Methodology*** Man behandelte 17 kongenitale, 41 neuropathische, und 15 infantile Skoliosen. Das durchschnittliche Operationsalter bei 63 Mädchen und 10 Buben betrug 3.5 Jahre (3–11). Die Korrektur der Verkrümmung wurde nach Cobb bestimmt. Komplikationen wurden evaluiert, ein Patientenzufriedensheits Score und das Wachstum der Kinder bestimmt. 21 der 73 Kinder hatten bereits eine operative Behandlung.
***Result*** Die primäre Krümmung betrug 68° (40°–145°). Nach der ersten Korrektur betrug die Krümmung 28° (10°–79°). Keine Komplikationen traten während der primären Implantation auf. Die Operationszeit betrug 154 min (110–215). In 15 Fällen wurde nur die konkave Seite instrumentiert, in 58 Fällen beide Seiten und in 18 Fällen zum Becken instrumentiert. Der Blutverlust betrug während der ersten Operation im Durchschnitt 285 ml (65 bis 450). Zwei der Patienten zeigte eine verzögerte Wundheilung, zwei andere Patienten entwickelten eine Pneumonie, die mit adäquater Therapie ausheilte.
In 4 Fällen kam es zu einem Stabbruch bei einseitiger Instrumentation, in 6 Fällen zu Schraubenbrüchen mit einem Durchmesser von 3.5 mm. In 10 Fällen mußten wir die Instrumentation verlängern.
Kein Infekt oder neurologische Komplikation trat in den 384 chirurgischen Eingriffen mit dem wachstumslenkenden Implantat auf.
In allen Fällen führten wir ein Monitoring mit SEP und MEP durch. Der durchschnittliche Krankenhausaufenthalt betrug 21 Tage (10 bis 32). 48 von den 73 Patienten hatten 1 bis 15 Nachspannungsoperationen. Die durchschnittliche Korrektur betrug 17.9° (22 %). In 18 Fällen mußte die sekundäre Krümmung nachinstrumentiert werden. Alle Patienten waren zufrieden und würden die Operation noch einmal durchführen lassen. In 18 Fällen führten wir bereits eine endgültige versteifende Operation durch.

***Spine V7.04***

### *Case Series of Five Children with Symptomatic Intraspinal Exostoses*

Hißnauer, T.*[1], Hagemann, C.[2], Ridderbusch, K.[1], Rupprecht, M.[1], Stücker, R.[1], Kunkel, P.*[2]

[1]Altonaer Kinderkrankenhaus, Kinderorthopädie, Hamburg, Deutschland, [2]Altonaer Kinderkrankenhaus, Pädiatrische Neurochirurgie, Hamburg, Deutschland

***Question*** Until today only few studies systematically investigated spinal involvement in patients with multiple hereditary exostoses (MHE). Spinal involvement was reported to be present in 70 % of MHE patients. In 25 % of patients exostoses protrude into the spinal canal. 75 % of these patients were previously reported to be asymptomatic and neurological intact.
***Objective*** We report here our experience with MHE patients harboring intraspinal lesions.
***Methodology*** We retrospectively reviewed our clinical charts of the last 6 years and identified 6 children with intraspinal lesions with a median age of 14.4 years (range 8–17 years). In all cases imaging studies with MRI were performed preoperatively.
***Result*** In our center 5 out of 6 patients with intraspinal lesions displayed neurological symptoms and were therefore operated. In 4 cases the intraspinal exostoses were located in the cervical spine (C3, C4, 2x C5), in one case in the thoracic spine (T10). Two lesions originated from the lamina, one from the facet joint, one from the spinous process and one ventrally from the vertebral body. All of them were either encroaching nerve roots (2 cases) or directly compressing the myelon (3 cases). Consequently two children presented with radiculopathy (C5, C6) and three with signs of myelopathy. We carried out 6 operations in these 5 patients, in one case the removal of the lesion was insufficient and had to be completed in a second intervention. In all cases a microsurgical approach was utilized to minimize surgical trauma. In all patients preoperative neurological symptoms resolved shortly after surgery.
This is the largest series of neurological symptomatic surgical cases of intraspinal exostoses in children. In our experience neurological impairment is present in more than 80 % of MHE patients with intraspinal lesions in this age group, which is higher than previously reported. Due to the possibly irreversible nature of neurological sequelae a timely detection of intraspinal exostoses is desirable to enable early surgical resection. Therefore, all patients with MHE should receive MRI imaging at least once during their growth period.

## Neuroorthopaedics 2

***Neuroorthopaedics 2 V8.01***

### *Kinematics During Stair Climbing in Cerebral Palsy*

Krautwurst, B.K.*[1], Dreher, T.[1], Wolf, S.I.[1]

[1]Universitätsklinikum Heidelberg, Klinik für Orthopädie und Unfallchirurgie, Heidelberg, Deutschland

***Question*** Patients with cerebral palsy show different gait patterns when walking on even ground as crouch gait (lack of knee extension)

or stiff gait (inadequate knee flexion) which are connected to different compensation mechanisms [1]. Patients with CP and with stiff gait showed a reduced toe clearance when walking on the even ground as compared to healthy controls. During walking on uneven ground the knee and hip flexion were increased and connected with this also the toe clearance [2]. However, it is unclear how these patients climb stairs.

***Objective*** The aim of this study was to evaluate the ability for stair climbing in patients with CP and to describe possible compensation mechanisms.

***Methodology*** Five patients (mean 16 years) with bilateral spastic cerebral palsy (GMFCS I–II) and five healthy controls (mean 27 years) underwent a clinical examination and an instrumented 3D motion analysis using a Vicon system. All subjects walked on even ground and climbed five steps up and down. Kinematics in the frontal plane as well as the step frequency were analysed by unpaired T tests.

***Result*** The patients climbed stairs slower than healthy controls (1.3 stairs compared to 1.7 stairs per second). During climbing upstairs the patients showed a decreased maximum dorsiflexion compared to the controls ($p = 0.03$) to lift their foot onto the next step. To get more toe clearance they had to increase their knee flexion (minimum knee flexion $p = 0.00$) and hip flexion to keep an upright trunk (mean trunk tilt $p = 0.59$) and pelvis (mean pelvic tilt $p = 0.11$). Going downstairs we found also a decreased maximum dorsiflexion in loading response at the next lower step in the patients group ($p = 0.01$). Therefore the patients had to flex their knees earlier to get to the next lower step.

In comparison between walking on even ground and stair climbing the patients showed decreased ankle dorsiflexion and increased knee and hip flexion compared to the controls. The trunk was further posteriorly tilted during level walking and going downstairs, whereas in walking upstairs the trunk position was similar to controls. There may be different compensation mechanisms during stair climbing and the walking patterns might be classified differently to level walking. This will be monitored in a larger patient cohort in the future.

***Neuroorthopaedics 2 V8.02***

### *Functional Improvement After Selective Dorsal Rhizotomy in Children with Bilateral Spastic Cerebral Palsy*

Funk, J.*[1], Panthen, A.[1, 2], Claus, N.[2], Wagner, C.[3], Haberl, H.[4], Lebek, S.[1]

[1]Centrum für Muskuloskeletale Chirurgie - Charité Universitätsmedizin Berlin, Kinder- und Neuroorthopädie, Berlin, Deutschland, [2]Charité - Universitätsmedizin Berlin, Sozialpädiatrisches Zentrum, Berlin, Deutschland, [3]Sana Klinikum Lichtenberg, Sozialpädiatrisches Zentrum, Berlin, Deutschland, [4]Charité - Universitätsmedizin Berlin, Pädiatrische Neurochirurgie, Berlin, Deutschland

***Question*** Selective dorsal rhizotomy (SDR) reduces spasticity and improves function in children with spastic cerebral palsy. The clinical relevance of the improvement is discussed controversely.

***Objective*** The aim of this prospective cohort study is to analyze the results of SDR with regard to gross motor function.

***Methodology*** Children who underwent SDR and were able to perform a gross motor function measure test (GMFM-88) preoperatively and at least 12 months postoperatively were included in this study (GMFCS I–III). Spasticity was assessed with the modified Ashworth scale (MAS), muscle strength with a manual function test (MFT), and passive range of motion (ROM) according to the neutral-null-method. Normally distributed continuous data as GMFM and ROM were analyzed with the T test for paired variables. Ordinal data such as MAS and MFT were evaluated with the Wilcoxon signed rank test. Bonferroni correction was applied when necessary.

***Result*** Between 2008 and 2011 74 SDRs were performed. 54 children with a mean age of 6.9 (±2.9) years underwent 12 and 24 months follow-up examinations.

MAS decreased significantly after SDR by about one grade ($p < 0.001$). GMFM improved significantly from 79 to 84 % 12 months after SDR ($p < 0.001$) and another 2 % between 12 and 24 months ($p = 0.002$). Considering only the target dimensions standing and walking function improved significantly by 9 % in the first 12 months ($p < 0.001$) and another 5 % in the second 12 months ($p < 0.001$) of follow-up. Muscle strength did improve significantly concerning knee extension ($p = 0.008$) and foot dorsiflexion ($p = 0.006$) by more than one grade 12 months after SDR. The strength of other lower extremity key muscles did not change significantly. ROM improved significantly by 6° concerning hip abduction ($p = 0.002$) after 12 months and foot dorsiflexion after 24 months ($p = 0.009$), but did not change significantly in the other dimensions of hip, knee and ankle passive movement.

Selective dorsal rhizotomy (SDR) increases motor function performance in children with spastic cerebral palsy by the reduction of spasticity if applied wisely. Clinically relevant changes in the GMFM and its target dimensions were achieved with SDR. The reduction of spasticity does not lead to a weakening of the muscles of the lower limbs. The positive effect of SDR on function does not deteriorate within the first 2 years after the procedure. Further analysis is necessary to evaluate the changes in muscle performance more functionally and to determine variables that predict the outcome of SDR more precisely.

***Neuroorthopaedics 2 V8.03***

### *Effect of Selective Dorsal Rhizotomy in Patients with Bilateral Cerebral Palsy GMFCS I/II*

Babin, K.*[1], Kunkel, P.[2], Hagemann, C.[2], Determann, K.[1], Brich, T.[1], Stücker, R.[1]

[1]Altonaer Kinderkrankenhaus, Kinderorthopädie, Hamburg, Deutschland, [2]Altonaer Kinderkrankenhaus, Pädiatrische Neurochirurgie, Hamburg, Deutschland

***Question*** Selective dorsal rhizotomy (SDR) is a neurosurgical spasticity reducing intervention for children with cerebral palsy.

***Objective*** The aim of this study was to explore the results of the first 14 children underwent SDR at the Altonaer Children's Hospital in Hamburg.

***Methodology*** 11 children with bilateral cerebral palsy GMFCS I/II participated at this retrospective follow up study. The children were operated at a mean age of 5 years and 7 months and were followed up until at mean 3 years after surgery (range 2–5 years, 2 months). The following measurements were performed before SDR and at follow up: Video analysis. Muscle tone and muscle strength was assesed in hip flexors, hip adductors, knee flexors and extensors and plantar flexors. Spasticity was measured with the Asworth scale. Passive range of motion (ROM) in hip, knee and ankle joint was measured with a goniometer. Motor function was assed using the cross Motor function score and from the functional assessment questionnaire (FAQ). Bony deformities and additional operative treatment after SDR were also recorded.

***Result*** reccurence Muscle Tone: At follow up muscle tone in the evaluated muscles was normalized in 9 children. 2 children showed a persistent mild spasticity in adductors and plantar flexors MAS1. In one child spasticity of the plantar flexors was evident MAS1. ROM: in hip, knee and ankle joint was unchanged or better in 7 children.

Two children develop a mild knee flexion contracture on both sides. In another two children shortening of the calf muscles worsen in comparison to the preoperative measurements. Muscle strength: 9 children showed unchanged good muscle strength. In two children decreased muscle strength in the knee extensors with mild crouch gait was evident. One of these patients showed additional weakness of the gluteaus medius. Bony deformities: 6 patients develop pes planovalgus deformity after SDR. In 3 patients pes planovalgus deformity worsen after SDR. In 4 patients with pes planovalgus deformity surgical correction was performed. Motor function: The mean FAQ changed from 7.9 preoperative to 9 postoperative. Eight children improved, three were unchanged, no worsen.
***Conclusion*** The majority of our patient had improved motor function after the SDR. Most challenging is to select the ideal patient for SDR to avoid muscle weakness. According to the literature bony deformities occur frequently in our study group. Despite the normalized muscle tone the patient after SDR still require additional treatment.

***Neuroorthopaedics 2 V8.04***

#### *Children with Spastic Hemiplegia and Bilateral Involvement: Do They Require Uni or Bilateral Management?*

Armbruster, M.[1], Böhm, H.*[1], Hösl, M.[1], Döderlein, L.[1]

[1]Orthopädische Kinderklinik, Kinderorthopädie, Aschau i. Chiemgau, Deutschland

***Question*** Categorization of cerebral palsy in uni and bilateral has shown good reliability. However the distinction can still be blurred since many children with unilateral CP may also have some degree of motor involvement on the opposite side. Therefore, it is not clear if they could be classified and treated as uni or bilateral.
***Objective*** The aim was to describe gait patters in hemiplegia with bilateral involvement and compare them to children with exclusively uni and bilateral involvement.
***Methodology*** In a retrospective study 25 patients with hemiplegia and bilateral involvement (4/25 GMFCS I), 51 patients with spastic bilateral CP (33/51 GMFCS I), and 53 patients with hemiplegia (41/53 GMFCS I) were included. All patients were able to walk barefoot without assistance (GMFCS I and II), between 4 and 16 years of age and did not had any surgeries. All patients underwent a 3D gait analysis followed by a clinical exam. The gait pattern of hemiplegia and hemiplegia with bilateral involvement were classified into 4 types according to Winters (1 foot drop 2 equinus, 3 knee and 4 hip involvement) The gait profile score (GPS, root mean square difference between 9 gait curves) describes gait deviations. For all 25 hemiplegia with bilateral involvement the GPS with respect to hemiplegia and bilateral CP were calculated and was tested by a paired t test.
***Result*** The classification revealed that 76 % of hemiplegic patients with bilateral involvement were type 3 and 4, whereas in hemiplegia it was only 37 %. The overall GPS was not significant different with respect to uni or bilateral patients. At the individual joint level pelvic tilt on the involved side was significantly closer to bilateral than to unilateral patients ($p = 0.03$) and pelvic rotation was closer to unilateral patients ($p = 0.04$). Summarized hemiplegic patients with bilateral involvement had more severe gait deviations than unilateral patients including the knee and hip joints. This might explain why pelvic tilt was comparable to bilateral patients. However, the pelvic retraction on the involved side agrees with the typical unilateral patients. In conclusion the gait pathology in hemiplegic patients with bilateral involvement cannot be clearly distinguished from the uni or bilateral group. We suggest that bilateral classification might be appropriate because both sides were involved. But pelvic retraction was comparable to hemiplegia, so that for transverse plane rotations treatment strategies for hemiplegic patients might be applied.

### Poster-Presentation 14.03. 15.03

***Poster-Presentation 14.03. 15.03. P.02***

#### *The Surgical Treatment of Non-idiopathic Spinal Deformity: Better Results in Younger Patients?*

Lattig, F.*[1], Kurz, S.[1], Reichel, H.[1], Weckbach, S.[1]

[1]Orthopädische Universitätsklinik am RKU Ulm, Orthopädie, Ulm, Deutschland

***Question*** Surgical treatment of non-idiopathic deformities is a challenging procedure. Robust evidence to define the optimal patient's age for surgery and the type of correction technique is still lacking and current practice is based on expert's opinion.
***Objective*** In this study we assessed the hypothesis that surgery in younger patients leads to better results.
***Methodology*** We conducted a retrospective analysis of a German university orthopaedic hospital database during a 3 year study period (2010–2013). Included were all patients surgically treated for non-idiopathic scoliosis and kyphoscoliosis. Outcome parameters consisted of the correlation of the patient's age and the postsurgical correction.
***Result*** Thirty-three consecutive patients surgically treated by the first author could be included in the study. 18 patients had a scoliosis and 15 patients had a kyphoscoliosis. Mean age was 14.6 years by the time of surgery (range 3–37 years). 7 patients have had surgical treatment for their deformity before elsewhere. Cobb angle of the main curve of scoliosis was 88° with a range from 20° to 158° and of the kyphosis 89° (range 62°–160°). Post surgically the Cobb angle was 37° and 50° respectively reflecting a mean correction of 52 % in scoliosis and 40 % in kyphoscoliosis. A correlation between correction potential and age could not be detected.
Based on our data the patients age is not the predominant factor of the potential of surgical correction in non-idiopathic spinal deformities. However, the remaining angle of deformity after correction in older and preliminary treated patients is higher and their risk might be increased due to prolonged surgical procedures.

***Poster-Presentation 14.03. 15.03. P.03***

#### *Osteoblastoma in the Paediatric Spine: Surgical Management*

Ruf, M.*[1], Mathis, N.[1], Smiszek, F.-G.[2], Pitzen, T.[1]

[1]SRH-Klinikum Karlsbad-Langensteinbach, Spinenchirurgie, Karlsbad, Deutschland, [2]Ernst-Moritz-Arndt Universität Greifswald, Klinik für Orthopädie und Orthopädische Chirurgie, Greifswald, Deutschland

***Question*** Beside the long bones, the spine is the most common localisation for osteoblastomas. They account for about 5 % of spinal tumors. It is a benign tumor, however, may grow expansively with local destruction and compression of neural structures. The leading symptoms are resistant local pain, loss of function, and deformity like torticollis or scoliosis. Surgical therapy is usually required.
***Objective*** To evaluate the outcome of surgical therapy of this rare entity, the following study was conducted.
***Methodology*** From 2003 to 02/2013, 45 children with surgical therapy of spinal tumors were identified in our databank. Osteoblastoma was histologically verified in 8 patients. One of them was lost for follow-up; seven children were included in the study. Mean age was 11 years 9 months (7 years 8 months–17 years 11 months). Localisation was most frequent at the cervical spine (4 pat.: C1, C2, C5, C6), one in the thoracic spine (T6), one in the lumbar spine (L3), and one at the sacrum (S1). All patients were treated surgically. Last follow-up with clinical and radiographic examination including CT or MRI was 3 years 8 months (9 months–7 years 8 months) postoperatively.
***Result*** A complete resection of the tumor could be achieved in all cases. The approach was posterior only in 3 cases, combined anterior-posterior in 4 cases (one transoral). Titanium mesh cages for reconstruction were used following corpectomy in 4 patients, and after resection of a lateral mass of C1 in one patient. Average 1.9 (0–6) segments were fused. In one case (osteoblastoma in the odontoid) a temporary instrumentation C1/C2 was removed after bony remodelling of the dens to regain atlanto-axial mobility. There was one complication: wound dehiscence at the softpalate requiring secondary suture.
At latest follow-up 5 patients were completely pain free, 2 patients reported occasional slight pain. There were no neurologic deficits. Radiographic examination revealed no tumor reccurrence in any case. There was no dislocation of the implants; bony fusion was already evident in 4 cases.
***Discussion*** The results of this study suggest that surgical treatment of paediatric spinal osteoblastoma is a safe and efficient procedure. Surgery results in a rapid and continuing pain relief. The surgery should strive for complete resection to avoid local recurrence of the tumor. Stability has to be restored with fusion as short as possible to minimise loss of function.

***Poster-Presentation 14.03. 15.03. P.04***

### *Treatment of Juvenile Foot Deformities with External Fixator*

Schmidt, M.*[1]

[1]Waldhofzentrum Kronberg, Kinderorthopädie, Kronberg, Deutschland

***Question*** The treatment of juvenile foot deformities is often very difficult and needs special techniques.
The use of external fixation allows the teatment of neglected complex foot deformities as clubfoot, cavusfoot and combined foot and leg deformities with a three dimensional stepwise correction.
***Objective*** Goal of this presentation is to present our results of 62 consecutive treated juvenile patients with acquired and often neglected unilateral or bilateral foot deformities with the use of a special external fixaton system with special hinges which allow the correction of even very severe deformities with our without tendon transfers ot osteotomies.
***Methodology*** All 62 patients who underwent a complex treatment of an neglected aquired foot deformity were seen for a follow up 3–6 months after end of the treatment period.
Forty-seven pateints had aquired foot deformities as neglected clubfoot, cavus foot or sequelae of polymyelitis, the rest were posttraumatic or neurogenic foot feformities often related with severe scaring.
All patients were treated with the used of a special designed external fixator allowing full correction of the deformity of the foot or if needed with combined treatment of the lower limb.
Treatment time with the fixator were between 2 and 9 months depending on the severity of the deformation, all patient were given a special orthosis after removal of the fixator.
***Result*** As result in all but one case we could achieve a plantigrade foot and correction of leg lenght discrepancy.
The feet often were very stiff but all patients could wear normal shoes with an orthosis or arch support.
One young patient from Angola with complex scaring after burn accident and multiple skin grafting could not be corrected due to and extreme bony and soft tissue stiffness and recurrent infections and an amputation was recommanded.
In general the method gives a high chance of good results in the treatment of complex neglected juvenile foot deformities, when a closed reduction is not possible. The young patients were often first time able to wear normal shoes and to walk plantigrade.

***Poster-Presentation 14.03. 15.03. P.05***

### *Spectrum and incidence of Anomalies of foot in Fibular Hemimelia*

Dussa, C.U.*[1], Paulitsch, F.[1], Döderlein, L.[1]

[1]Orthopädische Kinderklinik, Kinderorthopädie, Aschau i. Chiemgau, Deutschland

***Question*** Are Synostosis always a feature of Fibular Hemimelia? How often do they occur? Does the extent of Synostosis/deformity correspond to the degree of shortening?
***Objective*** The purpose of the study is to report the spectrum and incidence of various deformities/abnormalities in foot with fibular hemimelia.
***Methodology*** A retrospective study was carried on patients with fibular hemimelia. Inclusion criteria were: Children above 10 years, presence of AP and Lateral X-rays of feet. Exclusion criteria were: bony surgery on the foot before the appearance of the bones.
Apart from patient demographics, the presence or absence of ball and socket joint, various coalitions, missing bones, Hypoplasia and Syndactylie, duplications, missing rays and bones, Abnormal Physis were documented. The missing metatarsals were identified comparing the metatarsal ratios with the metatarsal ratios in the normal feet.
***Result*** One-hundered and eight Fibular hemimelia patients were indentified on the database of which only 65 patients were included in the study based on the inclusion criteria. Male: Female ratio was 48:17.
Abnormality Number of feet
Absence of fibula 45
Ball and socket joint 20
Talo-Calcaneal Fusion 65
Talo-Navicular Fusion 52
Calcaneo-cuboid Fusion 55
Cuneiform-Cuneaform Fusion 7
Naviculo-Cuneaform Fusion 16
Toe Apalsia 2
Soft tissue Syndactyly 10
Bony Syndactyly 2
Toe and Metatarsal Duplication 5
Metatarsal Hypoplasia 12
MT Growth plate duplication 19
Feet which had 5 rays had no other bony coalitions apart from Talo-calcaneal coalitions. All 3 cuneiform bones were seen only in 17 patients. And all of these feet had either 5 or 4 rays. Comparison of metatarsal ratios showed the missing ray to be the 5th Metatarsal. In 43 feet the either the intermediate or the lateral cuneiform (C2 or C3) was missing. In these feet, the missing metatarsal corresponded either to the second or the third in most cases. In feet with only 2 rays, both the intermediate and lateral cuneiform were absent. Most common growth plate abnormality was its presence at the distal end of 1st Metatarsal. Rarely an accessory growth plate was also present at the proximal end of 2nd Metatarsal. Although the ball-and-socket ankle joint was common in legs where fibula was present, 33 % of the ball-and-socket ankle joints had absent fibula.
As the name indicates, in fibular Hemimelia the deformities do not confine themselves to the lateral side of the foot.

***Poster-Presentation 14.03. 15.03. P.06***

### *Pedobarographic Results of Clubfeet After Peritalar Release Surgery*

Lebek, S.*[1, 2], Gutteck, N.[3], Panian, M.[3], Wohlrab, D.[3]

[1]Charité- Universitätsmedizin Berlin, CMSC, Kinder- und Neuroorthopädie, Berlin, Deutschland, Berlin, Deutschland, [2]Charité-Universitätsmedizin Berlin, CMSC, Kinder- und Neuroorthopädie, Berlin, Deutschland, [3]Orthopädische Universitätsklinik, Halle, Deutschland

***Question*** In order to assess the results after surgical clubfoot correction there exist several scores using primarily clinical and radiological data. Since the introduction of the Ponseti method X-rays are taken only infrequently. The function of the feet gets more into focus and thus, the dynamic pedobarography is used to evaluate pathological feet more routinely in adults.
We wanted to know whether it could be an appropriate instrument for assessing children's feet.
***Objective*** We wanted to know which pedobarographic patterns are shown in extensively operated feet and which differences in dynamic pedobarography exist between those and normal feet.
***Methodology*** Eleven children (7 boys, 4 girls) with 16 operated clubfeet (peritalar release) were evaluated by dynamic pedobarography. The results were compared to those of an age matched control group. The surgery was performed on average 8.3 years (5–12 years) before the evaluation. The average age of the clubfoot group was 9.1 years (SD 3.3.) and of the control group 9.0 years (SD 3.3 years). The pedobarography was done using the Emed system, Novel, Germany. The statistical analysis was performed using SPSS (Mann–Whitney test, $p < 0.05$).
***Result*** In the clubfoot group the time force integral and arch index were significantly higher (FTI total p 0.016; AI p 0.022). There were no differences in maximal power, foot progression angle or the geometrical indices.

***Poster-Presentation 14.03. 15.03. P.08***

### *Flexor Hallucis Longus Dysfunction: an Underestimated Problem?*

Lamprecht, E.*[1]

[1]Kantonsspital Winterthur, Kinderorthopädie, Winterthur, Schweiz

***Question*** Problems and dysfunction of the flexor hallucis longus tendon—like tendinosis, stenosing tendosynovitis, triggering and partial atraumatic ruptures—are often unrecognized or misdiagnosed. The disorder is better known in ballet dancers or athlets, but in the literature rarely mentioned among nonathletic patients.
The most constant clinical sign—besides tenderness and pain around the medial ankle—is a relative shortening of the flexor hallucis longus tendon. Our question is, if an early diagnosed “hallux rigidus” with its limited dorsal extension is much rather a clinical symptom of stenosing tenosynovitis of the FHL tendon and leads only secondary to a constant stress onto the cartilage of the first metatarsophalangeal joint.
***Objective*** We strongly point out to this often unrecognized disorder—to prevent the patients from long lasting unnecessary and unsuccessful therapies other than surgical decompression of the fibroosseous canal of the FHL tendon.
***Methodology*** We report datas of 15 patients (19 feet), the different signs and symptoms, the diagnostic evaluation and tests as well as the clinical and objective findings to establish the correct diagnosis. In two patients the tendon problem was overlapped by a proved tarsal tunnel.
Indications for the operative procedure are listed and the operative technique and after treatment is described.
All patients were treated with surgical tenolysis of the FHL tendon from its fibroosseus canal.
***Result*** In established stenosing tendiosynovitis of the flexor hallucis longus tendon, conservative treatment reveals unsatisfying results.
The outcome objectives were pain, mobility of the great toe (dorsal extension) and return to activities before symptoms occurred.
Following the anatomic conditions, the surgical procedure is simple and almost immediate pain relief and full function of the tendon is gained.

***Poster-Presentation 14.03. 15.03. P.09***

### *Pes equinus in Young Women: Psychogen Reasons have to be Considered*

Lengnick, H.*[1], Dussa, C.[2], Döderlein, L.[2]

[1]Behandlungszentrum Aschau, Kinderorthopädie, Aschau, Deutschland, [2]Behandlungszentrum Aschau GmbH, Orthopädische Kinderklinik, Aschau im Chiemgau, Deutschland

***Question*** There are different reasons for the development of pes equinus like cerebralpalsy, tumor, inflammation, habituell and others. In some cases the reason for equinus deformity remains potentially unclear. Psychogen interferences are sometimes not focused and have to be considered in the diagnostic advisement after excluding other somatic reasons.
***Objective*** Psychogen interferences are sometimes not focused and have to be considered in the diagnostic advisement after excluding other somatic reasons.
***Methodology*** We present the case of a17- and 14-year old girl presenting with pes equinus in our clinic:
Case 1: The patient presented with −5° dorsiflexion of the left structurell equinus foot. Discoloration of the skin and subcutaneous hematoma in the middle part of the calf was observed. The past medical history reflected a muscle lesion 2 years ago during sports accident. Ultrasound showed a small fibrotic area of the M.gastrocnemius, EMG, MRT ZNS and spine, angiography were without pathological findings. After gait analysis and examination under anaesthetic we performed Strayer procedure of M.gastrocnemius. Due to remaining pes equinus 8 weeks later percutaneuous Achilles tendon lengthening was done. Postoperatively she developed kind of spastic, leading to gliding of the foot within the cast causing pressure mark and sensibility deficit at the dorsum of the foot. Over a few weeks talipes calcaneus developed with ankle dorsiflexion of +40° despite limited lower leg orthosis. One year after OP she presented again with manuell not correctable pes equinus due to kind of spastic of the plantiflexors and again skin discoloration. Non-operative conservative treatment with only wearing heel crank, physiotherapy and acupuncture led to improved dorsiflexion of +5° after 4 weeks. Ambulatory psychosomatic clarification was interrupted by the patient.

***Result*** Reasons for pes equinus are multifunctional and have to be clarified carefully. Psychogen mechanisms and interference due to secondary positive effects of disease have to be considered especially in young girls.
Operative interventions have to be indicated very carefully, postoperative outcome might be worse. Therefore conservative treatment and psychosomatic clarification are useful methods to treat these patients.

***Poster-Presentation 14.03. 15.03. P.10***

### *Late Diagnosis in Tarsal Coalition*

Dornacher, D.*[1, 2], Reichel, H.[1, 2], Taurman, R.[1, 2]

[1]Universität Ulm, Deutschland, [2]Orthopädische Universitätsklinik am RKU Ulm, Orthopädie, Ulm, Deutschland

***Question*** Besides the rather seldom seen dense coalitions of the hindfoot in children with paraxial shortening of the lower limb (fibular hemimelia), tarsal coalition is a relatively common pediatric orthopedic disorder. The latter variety is typically asymptomatic until about the age of ten. Presumably, after that age, the ossifying foot becomes more rigid and is not longer able to compensate for the congenital tarsal coalition. The patients present with a fixed flat foot, often accompanied by a spasticity of the peroneal muscles.
***Objective*** Almost all of the patients have been over conservative treatment in vain, without having been diagnosed correctly.
***Methodology*** We report on 20 patients with a tarsal coalition, initially presented to our hospital between 2008 and 2012 because of a painful flat foot resistant to treatment. None of the patients received diagnosis other than "flat foot" or "haglund's syndrome", previously. The patients' history was taken comprehensively, including exact duration of symptoms and treatment already administered.
***Result*** Until final diagnosis, the shortest medical history was found in two young male with 6 months, the longest with 60 months in the oldest patient, mean medical history 33 months. For all except one patient multiple types of orthopedic insoles were prescribed, often for several years. In two young male patients the tibiotalar joint was injected repeatedly. One female received unsuccessful surgical treatment because of suspected "haglund's syndrome". To the time of the first presentation in our outpatient department, patients mean age was 16.8 years (range 10–25 years). After thorough diagnosis, the twenty-two tarsal coalitions split to eight calcaneonavicular (CN) and fourteen talocalcanear (TC). Pediatrists referred 22.5 months after onset of the symptoms, general practitioner referred after 36 months and orthopedists after 39 months.
Unfortunately, tarsal coalition is an often-overlooked orthopedic disorder. In unilateral, painful flat foot, tarsal coalition should be taken into consideration. With the aspect of a rigid hindfoot in the clinical examination, the suspected diagnosis can be supported. In bony coalition radiographic examination shows specific features: c-sign in TC and anteater sign in CN coalition. With MRI as the state-of-the-art diagnostic tool, fibrous or cartilaginous fusion can be detected. Since conservative treatment almost always is unsuccessful, generous excision and interposition of local muscle or fat graft is recommended.

***Poster-Presentation 14.03. 15.03. P.11***

### *Hip Dysplasia in MPS I: a MRI Study*

Breyer, S.*[1], Rupprecht, M.[1], Muschol, N.[2], Babin, K.[1], Stücker, R.[1]

[1]Altonaer Kinderkrankenhaus, Kinderorthopädie, Hamburg, Deutschland, [2]UKE, Pädiatrie, Hamburg, Deutschland

***Question*** MPS I is a disorder caused by the accumulation of glycosaminoglycans in all tissues. The syndrome is characterized by a variety of musculokeletal abnormalities. The main features of interest are hip dysplasia, kyphosis, genu valgum and stiff hands. Patients with MPS I get bone marrow transplantation since 1981, but still, as the patients mature, clinical and radiographic abnormalities of the hips develop. Life expectancy is increased, so the treatment of hip dysplasia is even more important. In our interdisciplinary work with the paediatric department we see a lot of patients with MPS I suffering of hip dysplasia. MRIs showed a better cartilaginous coverage of the head of the femur, than the plain radiograph. We were able to see the dynamic situation with intraoperative done arthrograms. In all cases the hip situation was more stable than expected from the radiogram, due to a good cartilaginous coverage of the head.
***Objective*** The above mentioned details showed a discrepancy in plain radiographs and the intraoperative situation. We want to describe the acetabular coverage of the femoral head in MRIs. There we can see the cartilaginous part of the acetabulum, missing in the plain radiographs. This information leads to a better preoperative planning. Until now there is no such evaluation described in literature.
***Methodology*** Twenty-eight patients are treated in our interdisciplinary clinic. In 12 children a plain radiological investigation of the hips was done. All of these patients underwent a bone marrow transplantation in younger age. Because of hip pain, 8 patients got a MRI of the hips. Plain radiographs and MRIs were assessed, and acetabular angle of Sharp and percentage of coverage of the head in the technique of Reimer were measured. In the MRIs we differentiate between the cartilaginous and the bone coverage.
***Result*** Children were in mean 7 years old. All patients demonstrated radiological signs of failure of ossification at the superolateral margin of the acetabulum and gradual distorsion of the femoral head with medial thinning. Mean acetabular angle in the plain radiographs was 35°, in the MRIs 38°. The percentage of bony coverage in the plain radiographs was 44 %. This could be verified in the MRI with a bony coverage in mean of 45 %. The cartilaginous coverage was surprisingly 72 % in mean. The soft tissue coverage of the femoral head seems to be 28 % more than the bony part. This explains the more stabile intraoperative situation.

***Poster-Presentation 14.03. 15.03. P.12***

### *Outcome of Modified Dega Acetabuloplasty for Congenital Dislocation in Developmental Dysplasia of the Hip Focusing on Internal and External Stabilization*

Vogt, B.*, Tretow, H., Schuhknecht, B., Horter, M., Schiedel, F., Rödl, R

Universitätsklinikum Münster, Kinderorthopädie, Deformitätenrekonstruktion und Fußchirurgie, Münster, Deutschland

***Question*** Treatment of congential dislocation (CD) in developmental dysplasia of the hip (DDH) is primarily conservative. If conservative methods fail surgical reduction of the hip is necessary. Acetabuloplasty (AP) is an established procedure for improvement of femoral head coverage. The surgical technique frequently has been modified. A hip spica cast is usually applied for at least 6 weeks after surgery.
***Objective*** This retrospective study evaluates the postop. results following modified AP on basis of clinical and radiographic parameters focusing on internal and external stabilization.

***Methodology*** Three-four children (m = 7, f = 27) with CD in DDH (Tonnis I = 3, II = 22, III = 12, IV = 8) treated by modified Dega AP using allografts (n = 45: r = 5, l = 18, b = 11) were investigated before surgery (age 30.0 m) and after a mean follow up (FU) of 40.1 m (age 5.8 years). Periop. parameters including time for surgery and fluoroscopy, complications and esp. techniques of internal (k-wire vs. no osteosynthesis) and external fixation (period of cast treatment) were evaluated. Pain level, ROM and gait were analyzed using Severin score. Measurement of established hip parameters, assessment of allograft consolidation and evaluation of complications such as femoral head necrosis (FHN) were done radiographically. U- and Fisher-test were used for statistics.
***Result*** Thirteen pat. required additional procedures like open reduction, femoral osteotomy or both. At FU all pat. were painfree. 4 cases had a leg length discrepancy (LDD). 2 of these pat. had a preexisting FHN before surgery. No new FHN occurred postop. Redislocation was found only once. Trendelenburg gait and limited hip ROM were seen in pat. with FHN and redislocation. The other 31 cases (91.2 %) had a Severin score A. After cast removal all pat. were mobilized under full weight bearing. Independent of casting period (6, 4, 3 weeks = 14, 13, 7) and osteosynthesis (k-wire = 7) all pat. had complete allograft consolidation and stable correction results. All radiographic parameters had significantly improved at FU. Revision surgery was necessary in 1 case with wound healing deficit. No other perioperative complications were found. Mod. Dega AP is a safe method with few complications even if performed bilaterally in a single operation that leads to good and (in medium-term) stable correction results. Additional procedures may be necessary to ensure a reliable reduction. On basis on our findings we consider, that there is no need for additional k-wire fixation. The casting period should not exceed 3–4 weeks.

***Poster-Presentation 14.03. 15.03. P.13***

### *Orthopedic Myth Hunters: Does Coxitis Fugax forecast a Morbus Perthes?: First Results of a Health Insurance-Data Based Study Design*

Stobbe, S.*[1], Filler, T.J.[2], Gödecke, S.[3], Lieb, A.[4], Placzek, R.[5]

[1]Actineo Personenschadenmanagement, Fachbereich Medizin, Köln, Deutschland, [2]Universität Düsseldorf, Klinische Anatomie, Düsseldorf, Deutschland, [3]Central Krankenversicherung AG, Controlling, Köln, Deutschland, [4]Orthopädische Praxis, München, Deutschland, [5]Universitätsklinikum Bonn, Klinik und Poliklinik für Orthopädie und Unfallchirurgie, Bonn, Deutschland

***Question*** Coxitis fugax (CF) is considered to be a frequent differential diagnosis of an early Perthes' disease (PD) [1, 2]. Whether this can be considered as predisposition for the occurrence of a subsequent PD, is for years a by both, pediatric orthopedics and pediatricians as well, controversial discussed question [3–5]. A typically demanded high level evidence prospective study design to clarify any correlation is hardly practicable with rarer diseases. By a cooperation with Central Krankenversicherung health insurance, it was possible to access data of N = 960 children with CF. The clinical studies available in the literature are between N = 41 [6] children with CF up to N = 160 [7].
***Objective*** To clarify the occurrence of PD in children who have previously had CF, the health insurance data was subjected to a standardized evaluation.
***Methodology*** This brand new way of data analysis is enabled since in the last years in Germany an increasingly structured collection of treatment data by the health insurers was started and allows the collection of meaningful numbers of cases for answering such related issues.
Statistical analysis was performed with a Pearson Chi square test, a singular sample with six characteristics was generated (Gender, disease 1, disease 2, age at disease 1 in life years, side of the disease 1, age at disease 2 in lifeyears).
***Result*** Of 960 children with at least one episode of CF occurred in 11 cases (1.15 %) in the later stages a PD. Children with two or more episodes of CF were more often, namely in 4 %, affected by PD. The mean time interval between the diagnosis was 160 days at a median of 36 days. The median is a stable parameter against outliers and therefore more meaningful here.
While the frequency of CF decreased with age continuously, for PD an age peak around the 5th life year was shown up, thus a direct dependency is not given.
All in all the data allows not the suggestion that PD is forecasted by CF. Thus, the data support the thesis of those authors who deny CF a role in the development of a later PD.
But: A reliable statistics can not be found at 15 cases of PD, it should be followed by the evaluation of the data of other health insurance companies, as already rolled out by our study group.

***References***
1. Manig M (2013) Der Orthopade
2. Nelitz M et al. (2009) Deutsches Arzteblatt
3. Landin LA (1987) JBJS Br
4. Mumme T et al (2005) ZFO
5. Wirth T (2006) ZFO
6. Mukamel M (1985) Clin ped
7. Erken EH et al (1990) JPO

***Poster-Presentation 14.03. 15.03. P.14***

### *Paediatric Hip Sonography at German University Hospitals: The Current Status*

Peterlein, C.-D.*, Fuchs-Winkelmann, S.

Universitätsklinikum Gießen und Marburg, Klinik für Orthopädie und Rheumatologie, Marburg, Deutschland

***Question*** The realisation of paeditric hip ultrasound, intern quality controls and training of the medical staff is different from clinic to clinic.
***Objective*** This study was conducted to evaluate the current status of hip sonography at German University Hospitals.
***Methodology*** A questionnaire was sent to all Head of Orthopaedic departments. They were asked to report on the technical way of hip ultrasound realisation, local organisation of consultation and options of advanced training for students and staff.
***Result*** The return rate of the questionnaire was 93.9 %. Paediatric hip ultrasound was performed at each University Hospital by staff of Orthopaedic/Trauma departments. We evaluated a well-established tradition of teaching both medical students and physicians on this topic. Training courses with focus on paediatric hip sonography are organised in 25.8 % of the German University Hospitals. In the majority of the asked Departments a 7.5 MHz linear transducer for ultrasound examination (93.5 %), a foot switch for rapid freezing of the ultrasound picture (93.5 %) and a cradle for positioning of the baby (100 %) is used. A guided probe device according to Graf is only used in 35.5 % of the asked clinics. Measurements of the sonograms is mainly performed on printed paper strips (61.3 %).
This survey documents the high value of paediatric hip sonography at German University Hospitals. Quality controls of the Orthopaedic Departments are carried out internally. For this reason both the implementation of standardised training courses and the schooling of well-trained instructors are mandatory.

**Poster-Presentation 14.03. 15.03. P.15**

### Combined Salter-Pemberton Pelvic Osteotomy

Hövel, M.*[1], Jäger, M.[1]

[1]Orthopädische Universitätsklinik Essen, Orthopädie, Essen, Deutschland

***Question*** Is it possible to improve the lateral roofing, the centering of the femoral head and the tuning between the volumes of the acetabulum and the femoral head by a modification of well-known pelvic osteotomies?

***Objective*** By the combination of anterior modified Pemberton osteotomy with dorsal pelvic osteotomy according to Salter we wanted to achieve a better lateral roofing, a safe centering of the femoral head and a tuning between the volumes of the acetabulum and the femoral head even in extreme dislocations of the femoral head in patients suffering of cerebral palsy.

***Methodology*** A total of 26 combined Salter-Pemberton pelvic osteotomies were performed in 25 patients from 1999 to 2008. The mean age at the operation was 13.0 years (7–27 years). The mean follow up was 4.8 years (2–9 years).

We measured the AC angle according to Hilgenreiner, the angle of the bearing plane according to Tschauner, the CE angle according to Wiberg and the ACM angle according to Idelberger and Frank in preoperative, postoperative and in the last available X-rays.

***Result*** The mean AC angle according to Hilgenreiner was preoperative 29.8° (SD 10.8°). By the operation the angle was improved to 22.2° (SD 6.5°). The angle of the bearing plane according to Tschauner in the last X-rays was by an average of 17.7° (SD 7.6°). Because of the extreme misalignment of the femoral heads the mean CE angle according to Wiberg was negative:

−16.1° (SD 28.5°). By our operations the CE angle was corrected to 18.1° (SD 5.4°). The last X-rays showed an additional improvement to 24.5° (SD 7.6°).

The mean distance of malalignment according to Idelberger and Frank was improved from 19.1 mm (SD 10.2 mm) to 8.0 mm (SD 2.1 mm). At late follow up the distance was 7.7 mm (SD 3.7 mm). The diameter of the acetabulum was reduced by the operation in the mean from 5.8 cm (SD 0.9 cm) to 5.4 cm (SD 0.7 cm). The results of our studies demonstrate that the combined

Salter-Pemberton pelvic osteotomy is a safe and effective procedure even in high grade dislocations of the hip joint caused by cerebral palsy.

**Poster-Presentation 14.03. 15.03. P.16**

### Forces, Growth Plate Areas and Compressive Stresses at the Proximal Femoral End

Heimkes, B.*[1], Eibl, D.*[1], Geith, T.[2], Günther, C.[1], Utzschneider, S.[1]

[1]Ludwig-Maximilians-Universität, Klinikum der Universität, Klinik und Poliklinik für Orthopädie, Physikalische Medizin und Rehabilitation, München, Deutschland, [2]Ludwig-Maximilians-Universität, Klinikum der Universität, Institut für Klinische Radiologie, München, Deutschland

***Question*** The proximal femoral end is particularly suitable for recognizing principles concerning the enchondral ossification and the longitudinal growth of the skeleton as it holds two morphologic identically structured growth plates of different size and different load.

***Objective*** Purpose of the study was to compare the forces, areas and stresses acting on the epiphseal growth plate and the greater trochanter growth plate during growth.

***Methodology*** Pseudonymised MRI-data of 36 hip-healthy children of different age and gender were taken to specify the loads, areas and compressive stresses on the epiphyseal and apophyseal growth plates. The areas were surveyed by means of the software tool OsiriX, the forces were calculated by established numerical modelings.

***Result*** 1. The areas of the two mentioned growth plates steadily increased from infancy until puberty. The epiphyseal growth plate increases 0.8 $cm^2$/year ($\sigma = 0.068$), the apophyseal growth plate 0.5 $cm^2$/year ($\sigma = 0.048$).

2. The epiphyseal growth plate area and the apophyseal growth plate area are at a ratio of 1:0.64. This ratio remains unchanged during the entire growth period ($\beta = 0.012$).

3. Both growth plates are loaded highly different during growth, but their computed compressive stresses are at a ratio of $\sigma_e$: $\sigma_a$ = 1:1.07 during the entire growth period ($\beta = -0.022$).

***Conclusion*** At least concerning the proximal femoral end you will find the fundamental skeletal remodeling law that growth plates of different size and different load are stressed by identical compressive stresses during the entire growth period. Increasing forces during growth enlarge the growth plates in order to keep compressive stresses constant.

**Poster-Presentation 14.03. 15.03. P.17**

### Surgical Treatment of a Large Desmoid Tumour of the Supraclavicular Region: a Case Report

Steiger, C.*[1], Beaulieu, J.Y.[2], La Scala, G.[3], Rougemont-Pidoux, A.-L.[4], Dhouib Chargui, A.[5], Lascombes, P.[1]

[1]Hôpitaux Universitaires de Genève, Service d'orthopédie pédiatrique, Genève, Schweiz, [2]Hôpitaux Universitaires de Genève, Unité de chirurgie de la main, Genève, Schweiz, [3]Hôpitaux Universitaires de Genève, Chirurgie pédiatrique, Genève, Schweiz, [4]Hôpitaux Universitaires de Genève, Pathologie clinique, Genève, Schweiz, [5]Hôpitaux Universitaires de Genève, Service de Radiologie, Genève, Schweiz

***Question*** Desmoid tumours are rare and benign neoplasms arising from fascial or musculoaponeurotic structures. Although this tumour does not metastasise, it displays an aggressive local behaviour, characterised by invasion and erosion of adjacent tissue.

***Objective*** We present a case of a nine-year-old boy with Klippel-Feil syndrome, cervical myelomeningocele and clavicular agenesis, who consulted our hospital for a large and painful cervical lesion.

***Methodology*** The boy had previously undergone surgery in the affected supraclavicular region in his country of origin, but unfortunately no detailed information concerning the surgery could be obtained. MRI and CT scan confirmed an 8 cm-diameter solid mass. Biopsy of the lesion revealed a desmoid tumour. As the local recurrence rate is very high after resection of these tumours, a conservative symptomatic treatment was started. Follow-up MRIs after 3 and 6 months showed a rapid increase in tumour size (to 12 cm in diameter), causing severe pain. Because of progression of symptoms, surgical treatment was indicated. To achieve complete tumour removal and best long-term results, a wide margin resection was planned. A multidisciplinary surgical team carried out the resection of the tumour, requiring partial resection of the brachial plexus (C5, C6 roots), resection of the humeral head and of the scapula. A latissimus dorsi with skin paddle rotation flap was used to cover the post-resection soft tissue defect.

***Result*** Histopathology confirmed complete tumour removal with >5 mm tumour-free margins. Patient recovery was uneventful with return to normal daily activities within 1 month. The 3 months follow-up MRI showed no evidence of tumour recurrence.
A multidisciplinary approach to this difficult tumour allowed for an optimal outcome for the patient.

***Poster-Presentation 14.03. 15.03. P.18***

### *Esthetic and Functional Improvement in PFFD (proximal femoral focal deficiency) with Deficient Hip Joint Development (Pappas type II–III) by Rotation Plasty Winkelmann Type BIII*

Exner, G.U.*[1], Feusi, A.D.[1, 2]

[1]Orthopädie Zentrum Zürich, Zürich, Schweiz, [2]Universität Zürich, Schweiz

***Question*** PFFD (proximal femoral focal deficiency) type II–III has no hip joint development. Winkelmann published one patient treated with his modification of rotation plasty by creating a new articulation by insertion of the lateral tibial plateau into the deficient acetabulum.
***Objective*** We present the results in three patients treated using this technique. They are compared to one patient, in whom this technioque was used after resection of the femur for an osteosarcoma, one patient with PFFD and complete reconstruction after a series of procedures and one patient, in whom rotation with fusion of the distal femur to the pelvis failed.
***Methodology*** At the time of rotation plasty patients (1 girls 2 boys) were aged between 4 and 10 years f/u is between 7 and 5 years after surgery.
All patients use their orthoprosthesis without needing any additional support (e.g. canes).
They are all highly satisfied and would chose the same treatment again. MSTS scores are presented.
***Result*** Rotation plasty is a technically highly demanding procedure, especially in such situations as PFFD, where the anatomy is distorted and structures as vessels and nerves are short.
However it is an option to improve orthoprosthetic fitting functionally and esthetically.

***Poster-Presentation 14.03. 15.03. P.19***

### *1 Year Follow up After AMIC Therapy in Osteochondral Necrosis of the Femoral Head in an 17 Year Old Football Player*

Wolfsteiner, J.*[1], Wagner, F.[1], Dingeldey, E.[1], Dullien, S.[1], Matussek, J.[1]

[1]Orthopädische Klinik der Universität Regensburg, Kinderorthopädie, Bad Abbach, Deutschland

***Question*** At the time of the first appointment there had already been pain in the youth's left hip joint for nearly 2 years. The MRI diagnostic showed a 4 cm diameter cartilage defect in the main weight bearing area of the femoral head. The morphology was untypical of Perthes disease.
***Objective*** Previously in our clinic there have been many AMIC and ACT therapies used for cartilage defects in knees and ankles giving good results. Due to the size of the defect and the collateral damage an OATS therapy would cause, we decided in favour of a trochanter flip osteotomy followed by a filling of the affected area with spongiosa from the left pelvis and an AMIC layer.
***Methodology*** Clinical diagnostics and MRIs were carried out post-operatively at intervals of 6 months. One year after the operation we carried out an additional motion analysis. Also, the Harris Hip Score was evaluated pre- and post-operatively at 12 months.
***Result*** During the course the follow up examinations the left hip was free of pain and showed a better range of motion. The motion analysis showed a homogeneous motion in comparison to our usual sport student grouping, with nearly symmetrical loading. The follow up MRI showed a good osteochondral integration of the transplant and a good remodeling of the femoral head. The postoperative Harris Hip Score was excellent. Currently, the patient is pain free and has recommenced soccer training.

***Poster-Presentation 14.03. 15.03. P.20***

### *Progressive Valgus Deformity of Distal Femur Following Supracondylar Osteotomy: Report of 3 Cases*

Dussa, C.U.*[1], Fischer, C.[1], Morvai, A.[1], Döderlein, L.[1]

[1]Orthopädische Kinderklinik, Kinderorthopädie, Aschau i. Chiemgau, Deutschland

***Question*** Review of 3 cases with progressive valgus deformity following a supracondylar osteotomy of distal femur.
***Objective*** The report presents an unusual complication following a relatively common surgery of distal femur.
***Methodology*** Supracondylar femur osteotomy is the one of the commonly performed surgeries in paediatric age group especially children with cerebral palsy. The osteotomy can be used to correct either a unidirectional or multidimensional deformities. Several complications such as damage to neurovascular structures, delayed and non-union, infection, under-correction and over-correction are known to occur. However a progressive valgus deformity with time following the surgery has not been described to our knowledge following this surgery. This occurs due to damage to the lateral growth plate while exposing the distal femur for plating. Radiological observation showed no bony bridge in all 3 cases. Therefore postulate a fibrous tether on the lateral side of the growth plate, strong enough to produce asymmetrical growth leading to the progressive valgus deformity.
At the time of detection of the progressive valgus deformity, more than 18 months have elapsed in all 3 cases. All 3 cases subsequently reoperated and a varus osteotomy of the distal femur was performed. In all 3 patients the osteotomy healed in the expected time without complication.
***Result*** The report highlights another potential complication of this surgery when proper attention is not paid to the growth plate at the time of surgery. There fore during the follow-up all cases have to be observed not only for the recurrence of the principle deformity but also progressive valgus deformity of the distal femur. Since no bony tether is typically seen, it is debatable if this deformity is amenable to correction with 8-Plate. We have no experience regards to this. It is also debatable if such tethers can be detected with radiological investigations such as MRI. Therefore clinical suspicion and anticipation of this complication is very necessary.

***Poster-Presentation 14.03. 15.03. P.21***

### *Successful Management of Acute Dislocation of the Hip in Down syndrome: A Case Report and Review of Literature*

Bröking, J.N.*[1], Vogt, B.[1], Schuhknecht, B.[1], Tretow, H.[1], Horter, M.[1], Schiedel, F.[1], Rödl, R.[1]

[1]UKM, Kinderorthopädie, Münster, Deutschland

***Question*** Down disease (DD) is associated with general hypermobility that can lead to clinically significant joint instability. Atraumatic dislocation of the hip (ADH) occurs in 5–14 % of patients usually between the ages of 2–10 years. However, in most cases ADH develops insidiously, whereas acute events are relatively rare. Connective tissue weakness, muscular hypotonia and the configuration of the pelvis and the proximal femur are controversially discussed as possible causes.
***Objective*** There is no particular treatment protocol in literature for patients with acute ADH in DD. Due to the mentioned pathoanatomic conditions a stable reduction of the hip over time is difficult to achieve and a challenging therapeutic target. This is a case report of a successful management of acute ADH in DD with discussion on the treatment performed to achieve a stable joint.
***Methodology*** A nine-year-old boy with DD was referred to our outpatient clinic. The day before he suddenly complaint about pain in the right hip while leaving his bed and subsequently was unable to walk. Clinically he presented with shortening and external rotation of the right leg. Plain radiographs showed an anterior ADH. No fracture or epiphysiolysis, but also no dysplasia of the hip or the proximal femur were found. Closed reduction of the hip was performed successfully without analgosedation and proved radiographically. For retention a hip spica cast in abducted position was applied for 3 weeks. There was no incidence of neurovascular damage before and after reduction. MRI study was done for exclusion of a vascular necrosis of the femoral head. To achieve a stable reduction with minimal risk of redislocation of the right hip triple osteotomy of the pelvis according to Tonnis, proximal femoral varus osteotomy and anterior capsular plication was conducted. After surgery a hip spica cast was applied for another 6 weeks.
In short-term follow up of 3 months a stable reduction without redislocation of the hip could be achieved. Clinically there was no evidence of instability. Full weight bearing was possible without pain or other complaints. Radiographically sufficient containment of the hip joint was documented.
***Result*** The outcome of atraumatic ADH depends on the time till primary reduction is reached, the direction of dislocation and concomitant damages. The presented therapeutic management led to good results with stable hip without any complaints in a short-term follow up. However, long-term assessment is necessary for final evaluation.

***Poster-Presentation 14.03. 15.03. P.22***

### *Modern Strategies in the Treatment of Proximal Femoral Focal Deficiency (PFFD) of Different Severity: Primary Prosthetic and Orthotic Treatment in Respect of the Pappas Classification—Chances and Limits*

Dingeldey, E.*[1], Matussek, J.[1]

[1]Orthopädische Klinik der Universität Regensburg, Kinderorthopädie, Bad Abbach, Deutschland

***Question*** Proximal femoral focal deficiency varies in severity from a short femur but normal hip joint up to severe cases without a hip joint and might cause extreme leg length discrepancies. Limb lengthening with distraction of callus will work up to approximately 10 cm.
The aim of further surgical realign treatment is to stabilize the hip and the leg for prosthetic fitting.
How can the treatment be adjusted to the different stages of skeletal development and individual needs?
***Objective*** The goal of treatment is to provide a compensation of leg length discrepancy to get an optimal function without pain and acceptable appearance as the patients want to take part in normal life activities.
***Methodology*** Surgical treatments are useful in Pappas classes IV–VII. In the last 12 years 10 children were treated from toddler's age onwards. They were observed every 3 months with a high compliance of their families.
***Result*** Six patients decided against surgery and obtained ortho prosthesis despite of a detailed instruction about the impact of surgical alternatives.
Four patients decided to do limb lengthening or improve the situation of the hip, which means derotation of proximal femur, transformation of femoral head or distraction of femoral callus
In the case of very short femurs the ortho prosthetic is a big challenge as the knee becomes the hip and must be put on a soft bearing.
Depending on social aspects the treatment has to be adjusted to the different stages of skeletal development and has to be tailored to particular needs.
Even in large leg length discrepancy, a good functional and cosmetic result can be achieved by ortho prosthetic treatment. But surgical intervention might be required to optimize fitting of prosthesis.

***Poster-Presentation 14.03. 15.03. P.24***

### *Diagnostic and Treatment Concept of Femoral and Tibial Torsion Disorders at the OKS*

Ellenrieder, B.*[1], Erschbamer, M.[1], Zdenek, K.[1], Wegener, R.[2], Payne, E.[1], Klima, H.[1]

[1]Ostschweizer Kinderspital, Kinderorthopädie, Claudiusstrasse 6, 9006 St.Gallen, Kinderorthopädie, St.Gallen, Schweiz, [2]Ostschweizer Kinderspital, Kinderorthopädie, Labor für Bewegungsanalyse, St. Gallen, Schweiz

***Question*** Torsion disorders of the lower extremity are common in infants and improve spontaneously in most cases during growth. Surgical correction is rarely indicated in cases where the extent of deformity persists outside the normal range and produce increased joint load as shown in the literature [1].
***Objective*** We present our experience for indications for correction, surgical treatment options and diagnostic methods. The treatment algorithm of the OKS (Ostschweizer Kinderspital) for decision finding and the treatment possibilities are described.
***Methodology*** In case of joint pain, functional disorders or gait deviation in combination with a deformity in bony torsion through physical examination (tibial torsion $<5°$ or $>40°$, femoral torsion $<5°$ or $>50°$) a CT scan is performed. If the CT scan confirms abnormal values [2] a 3D gait analysis will be performed. If the typical deviations in kinematics and kinetics are proved, a derotation osteotomy is considered [3]. We use for distal tibia and distal femur correction a percutaneous osteotomy and a unilateral external fixation system. An external jig is used for precise correction in tibia osteotomy. Approximately 1 year after removing the device 3D gait analysis is repeated for therapy control.
***Result*** If functional problems due to persisting torsion disorders in physical examination are confirmed by CT scan and gait analysis an operative intervention is indicated. Gait analysis is helpful to demonstrate the amount of deviations in kinetics and kinematics pre-interventional and the improvement of these parameters post-interventional.

***References***
[1] Sass P et al (2003) American Family Physican

[2] Liodakis E et al (2011) Springer
[3] Höglinger M et al (2010) Gait posture

***Poster-Presentation 14.03. 15.03. P.25***

### *Lengthening of Long Bone with Unilateral Fixator over Plate*

Dussa, C.U.*[1], Paulitsch, F.[1], Morvai, A.[1], Khavkin, E.[1], Döderlein, L.[1]

[1]Orthopädische Kinderklinik, Kinderorthopädie, Aschau i. Chiemgau, Deutschland

***Question*** Is lengthening over plate using a monolateral fixator (Orthofix) an alternative to ring fixator? Can the fixator-time be reduced significantly using the combination?
***Objective*** The purpose of the study is to assess the effectiveness of lengthening of long bone in lower limb using a Monorail Fixator over a Plate. The study also aims to look into the distraction time, healing index, and incidence of complications such as infection, fracture, delayed healing.
***Methodology*** Prospective data base was used in this study. Consecutive patients operated between 2010 until 2012 were included in the study. At the time of surgery, Orthofix Monorail system was used as a distraction device either in the Femur or Tibia. At the same time, a locking plate was implanted on one side of the Femur or Tibia following corticotomy. Following distraction the Fixator was explanted and the plate was fixed with locking screws in the distal fragment to stabilize the callus.
Patient demographics, etiology of limb length discrepancy, during of distraction, and fixator along with complications were documented. Inclusion criteria were: limb lengthening using a monolateral fixator over a plate.
Fifteen Patients whose limbs were lengthened using ring fixators were used as controls.
***Result*** Sixteen limbs were distracted using a monolateral fixator in the above mentioned time. Of these 4 were excluded as no plating was performed at the time of fixator application. Of the remaining 12, M:F = 7: 5. In all cases the index bone was lengthened to the set target of 5 cm. In 11 cases the fixator was removed within 3 months of completion of distraction. There was no case of delayed callus formation was seen. Bone grafting was not required in any case. No permanent contractures of Knee or Ankle were seen. The complications included: plate breakage (3), Pin-tract infection (1). In all 3 cases where the plate fractured, a Titanium Plate was used, and the fracture was through the unoccupied screw holes. Premature unprotected weight bearing was the cause of fracture in all the 3 cases. We have since then using a Stainless Steel Locking plate to bridge the callus and also closed the unoccupied screw holes with screw heads. Lengthening over plate is a good alternative to the conventional lengthening of long bones using a ring fixator. It is user and operator friendly. The complication rates in this series are patient dependent except the pin-tract infection.

***Poster-Presentation 14.03. 15.03. P.26***

### *Operative Treatment of Severe Skeletal Malformations of the Lower Extremity Caused by Phosphate Diabetes*

Arnold, C.R.*[1], Rogalski, M.[1]

[1]Helios Klinikum Berlin-Buch, Zentrum für Orthopädie und Unfallchirurgie, Berlin, Deutschland

***Question*** Phosphate diabetes is a rare disease with an incidence of 1 in 325.000. The manifestation occurs in the early childhood caused by a mutation of the short arm of the X-chromosome. The mutation is responsible for an increased renal phosphate excretion. This leads to a low level of serum phosphate and consecutively to severe skeletal malformations, growth delay and impaired hearing.
We present a case of a 12-year old boy with severe skeletal malformation of the lower extremity caused by phosphate diabetes. As a refugee from Angola he never received any previous treatment in his home country. He was presented by an international care organization in our hospital for diagnosis and evaluation of operative treatment options. The patient was barely able to move in a wheelchair or crawling. The lower extremity, both femurs and tibias showed a massive significant varus and antecurvation error with a poor muscular function.
***Objective*** Our goal was the improvement of mobility and pain reduction by conservative and operative treatment of his lower extremity.
***Methodology*** As an operative treatment of the skeletal malformations we performed multiple correction osteotomies and intramedullary stabilization with the telescopic "Fassier-Duval"® nails because of the still opened growth plate and narrow intramedullary cavity, which did not allow insertion of locking nails. Preoperatively the localization of the osteotomy based on the X-rays of the lower extremity was planned computer assisted.
***Result*** The correction of axial deviations of the lower and upper leg was done in a two step manner starting with the right leg. The postoperative treatment in our department was based on short cast immobilization, intensive physiotherapy with orthopedic aids like the posture walker, storage rails and crutches. After performance of the tibial osteotomies we decided to continue with correction of both femora. During the last outpatient evaluation we saw a great functional outcome postoperatively with significant better mobility and pain reduction. The postoperative close-knit X-ray controls of the lower extremity showed a good osseous rebuilding of the osteotomy zones. Three months postoperative the mobilization is possible with full weight bearing.
Adequate medical aid of refugee children from developing countries, who cannot be treated in their home country is an ongoing challenge for staff, sponsors and the patients.

***Poster-Presentation 14.03. 15.03. P.27***

### *Hamstring Lengthening for Therapy of Crouch Gait Might Lead to Muscle Weakness and Stiff Knee Gait in Patients with Cerebral Palsy*

Lengnick, H.*[1], Dussa, C.[2], Döderlein, L.[2]

[1]Behandlungszentrum Aschau, Kinderorthopädie, Aschau, Deutschland, [2]Behandlungszentrum Aschau GmbH, Orthopädische Kinderklinik, Aschau im Chiemgau, Deutschland

***Question*** Several studies report to significantly improve knee motion by operative hamstring lengthening in patients with spastic diplegia. Our clinical observation indicated several patients after hamstring lengthening with stiff knee gait showing an inefficient gait pattern, limited range of motion of the knee joint and reduced walking ability.
***Objective*** The aim of this study was to examine the effect of hamstring lengthening in patients with cerebral palsy.
***Methodology*** We studied a patient group of 31 patients who underwent hamstring lengthening due to crouch gait in cerebral palsy. Follow up time was 9.4 years after operative treatment. Patients were classified by gross motor function classification system (GMFCS). Clinical examination considered range of motion of the knee joint,

Duncan Ely-test for assessment of shortening/dysfunction of M.rectus femoris, patella position, strength of knee extensors and flexors using Medical Research Council (MRC) scale. Duncan-Ely test was modified by three steps of severity of buttocks elevation: level 1 = 5 cm, level 2 = elevation 10 cm, level 3 = elevation 15 cm.
In eleven cases gait analysis was postoperatively conducted to objectify pelvic position
***Result*** Knee extension ability showed a very slight trend to hyperextension (−0.5°) whereas knee flexion ability was slightly below normal value (117.1°). Duncan-Ely test was in all patients positive, on average level 2 was observable. Quadriceps strength was higher than power of hamstrings (MRC: 3.7 vs. 2.9). Patella position was in 79 % pathologic with positive Insall-Salvati Index >1.2 or patella palpation outlying patellofemoral sliding groove.
Seven patients showed stiff knee gait and pathologic internal knee flexion moment due to strength of M.quadriceps femoris in relation to weaker hamstrings.
***Conclusion*** Operative treatment of hamstring lengthening might result in weakening of hamstring power leading to overactivity of knee extensors. Stiff knee gait with energy consuming gait pattern might be the consequence. Therefore the indication for operative hamstring lengthening has to be made just as carefully as the procedure itself. Bony correction in structural knee flexion deformity, leaving muscles untouched, is a serious alternative.

***Poster-Presentation 14.03. 15.03. P.28***

### *Serial Casting and Redressement Therapy in Neuromuscular Diseases*

Ellenrieder, B.*[1], Erschbamer, M.[1], Zdenek, K.[1], Payne, E.[1], Klima, H.[1]

[1]Ostschweizer Kinderspital, Kinderorthopädie, Claudiusstrasse 6, 9006 St.Gallen, Kinderorthopädie, St.Gallen, Schweiz

***Question*** Serial casting and redressement therapy (1, 2) are established and well proven techniques particularly for pediatric joint contractures. Especially demanding are joint contractures in patients with Arthromyogryposis (AMC), Meningomyelocele (MMC) and neurological diseases. We present an overview of the literature and our concept of techniques and required infrastructure.
***Objective*** The different techniques of redressement consists of serial casting, orthoses with adjustable joints or external fixation systems.
***Methodology*** The literature shows that serial casting is most successful in the lower extremity and in early childhood (3). For the therapy of joint contractures in bilateral cerebral palsy a combination with botulinum toxin is superior in the long-term (4), as proven by gait analysis (5). For a successfull therapy a team of specialists and an adapted infrastructure is required. For the treatment of clubfoot deformity associated with AMC, MMC and other neuromuscular diseases, Ponseti therapy is applied but often not satisfactory. This therapy is mostly followed by surgical intervention and orthoses therapy during growth resulting in corrected, but stiff feet. Casting therapy is not always sufficient treating rigid joints such as AMC. An external fixation system (Ilizarov) combined with arthrolysis is necessary. We suggest an early onset of casting therapy in neuromuscular patients with joint contractures.
***Result*** Serial Casting and redressement therapy is an evidence based therapy for joint contractures, which show good results if a strict concept is followed and is supported by a well coordinated team and infrastructure. The addition of botulinum toxin, orthoses therapy or surgical intervention may be necessary. The children's age, the neurological disease, patients' and parents' compliance, technique and extent of the contracture influence the success of this therapy.

***References***
[1] Jain et al (2008) Indian J Pediatr
[2] Brouwer et al (1998) Dev Med Child Neurol
[3] Westberry et al (2006) J Pediatr Orthop
[4] Desloovere et al (2001) European J Neurol
[5] Thompson et al (1998) Dev Med Child Neurol

***Poster-Presentation 14.03. 15.03. P.29***

### *Molding Helmets: Experiences of >1000 Cases*

Willenborg, H.*[1]

[1]Orthopädie im Annastift Hannover/Orthopädie der MHH, Department Kinderorthopädie, Hannover, Deutschland

***Question*** Is helmet-therapy a method which is needed? Who should work with? Which preconditions should be fulfilled to treat the affected babies?
There are various causes for head deformities in infants.
But: we have never seen so many cases since inauguration of the "back to sleep" campaign to avoid the sudden infant death syndrome (SIDS).
The second USA-campaign "tummy time" did not reach Europe yet.
***Objective*** What can be reached realistically? We made a study including more than 1,000 cases. We can show, that helmet therapy is effective. But the better goal would be, preventing severe head-deformity by doing the correct things in the early months of life. It would be better, not only to give the instruction for newborns to lye supine while sleeping, but also to advice the parents to have them in prone position for a few minutes every day while being awake and watched. If the baby wants to sleep again it has to be put on its back.
Lying prone, the back-head can flatten because of the head's weight. The baby has to train the muscles of the arms, neck and body. Spine-stability and movement like crawling develops from prone position.
All disciplines that are involved in the treatment of little children with head deformities should be aware of the possibilities of this method and its limitations.
***Methodology*** In a short time-corridor in the second half of the first year of life we can guide the growth of the head with an orthosis. This brace prevents further growth of the dominant areas and offers space for the skull to expand.
Since the growth of the skull decreases at the end of the first year of life, the duration of treatment depends on the severity of the deformity and the age at the onset of therapy.
The circumference of the head increases by 12.5 cm in the first, but only by 2.5 cm in the entire second year of life.
Treatment times are between 2 and 8 months, in special cases probably longer than 1 year. The brace must be worn 23 h a day, the free hour is necessary to clean the helmet and the head.
***Result*** With helmet therapy we can have very good results, depending of several parameters: age of the baby, severness of deformity, compliance of the parents—applicating a well fitting product.
We saw better results in the end of helmet therapy beginning the therapy in younger age of the children. The average age of beginning in our department was 8 month (younger would be better!). The average of treating-time was 6 months. The time of treatment wasn't very much longer in older children.

***Poster-Presentation 14.03. 15.03. P.30***

### *Trampoline Related Injuries in Children. A Summary of 65 Cases*

Hißnauer, T.*[1, 2], Ridderbusch, K.[1], Sommerfeldt, D.W.[3], Rueger, J.M.[2], Stücker, R.[1], Rupprecht, M.[1]

[1]Altonaer Kinderkrankenhaus, Kinderorthopädie, Hamburg, Deutschland, [2]Universitätsklinikum Hamburg-Eppendorf, Klinik und Poliklinik für Unfall-, Hand- und Wiederherstellungschirurgie, Hamburg, Deutschland, [3]Altonaer Kinderkrankenhaus, Kinderunfallchirurgie, Hamburg, Deutschland

***Question*** Backyard trampoline jumping is very common and extremely popular among children of every age. We recognized a considerable increase of trampoline related injuries in pediatric patients presenting to the emergency department.
***Objective*** The ambition of this case review is to evaluate severity of the trampoline related injuries and it's circumstances.
***Methodology*** We searched through the medical reports including diagnostic imaging from the emergency department to analyze which children were presenting with backyard trampoline injuries. The observed time period comprised 18 months from February 2012 until August 2013.
***Result*** In the analyzed period of time 65 children were presenting with trampoline related injuries in the emergency department. The median age of theses children was 7.8 years (age range 2–17 years). 32 of the patients were boys, 33 were girls. Apart from less serious injuries, we also saw many cases of severe injuries. 29 (44.6 %) of the children had fractures, followed by a quantity of 24 (36.9 %) children with sprains and distortions. A number of 5 (7.7 %) of the infants suffered from cranial bruises or brain concussion. 4 (6.2 %) of the presenting children had a joint dislocation and 2 (3.1 %) soft tissue injuries caused by a trampoline accident. One boy (1.5 %) even broke his growing rod, an implant to treat juvenile scoliosis, jumping on a trampoline. Jumping on trampolines is certainly a high risk backyard recreation which frequently causes severe injuries.

***Poster-Presentation 14.03. 15.03. P.31***

### *Subfascial Closed-Suction Drains do not Reduce the Incidence of Surgical Site Infections in Musculoskeletal Hip Surgeries in Children*

Druschel, C.*[1], Heck, K.[2], Funk, J.F.[3], Placzek, R.[2]

[1]Charite - Universitätsmedizin Berlin, Centrum für Muskuloskeletale Chirurgie, Berlin, Deutschland, [2]University Hospital Bonn, Clinic for Orthopaedics and Trauma Surgery, Department of Pediatric Orthopedic Surgery, Bonn, Deutschland, [3]Centrum für Muskuloskeletale Chirurgie - Charité Universitätsmedizin Berlin, Kinder- und Neuroorthopädie, Berlin, Deutschland

***Question*** Surgical site infections cause excess morbidity and mortality in modern surgery. Insertion of closed-suction drains is one of the most common approaches to reduce their incidence. However when dealing with children, a benefit has not yet been proven, while handling and removal remain complicated.
***Objective*** We compared retrospectively two groups of children undergoing similar muscoloskeletal surgery with comparable osseous/soft tissue damage in order to evaluate the efficiency of wound drainage in children. Group 1 was postoperatively treated a subfascial surgical drain, group 2 two was left undrained.
***Methodology*** We analyzed 63 children receiving 75 surgical procedures (group 1) and 79 children undergoing 130 surgical procedures (group 2) in a dual center study. Both groups were compared concerning demographic data, pre-existing conditions, surgical indications/procedures, intraoperative complications and duration of hospitalization. Postoperative complications were classified according to the Clavien-Dindo grading system.
***Result*** Both groups displayed similar initial situations concerning age at operation, gender, pre-existing pathologies, surgical indications. In group 1 10.7 % of the patients received a solitary acetabuloplasty, 53.3 % a corrective femoral osteotomy. A combined procedure was performed in 36 %. In group 2 solitary acetuloplasties were performed in 26.9 %, femoral osteotomies in 22.3 % and combined procedures in 20 %. Implant removal took place in 30.8 %. No intraoperativ complications occurred in either group. In group 2 a significant shorter hospitalization time was recorded (13.7 ± 9.3 vs. 5.7 ± 2.7 days). A follow-up of 19.4 ± 12.3 months in group 1 observed 31 complications, follow-up of 30.6 ± 24.7 months in group 2 depicted 26 complications ($p < 0.001$). The majority of postoperative complications consisted of lack of surgical success in both centers. Both groups revealed mainly I/II complications. Grade III complications were recorded in 3.2 % for group 1 and in 11.5 % for group 2. No grade IV and V complications were reported. Intra-/postoperative requirement of blood transfusion amounted to 6 units of packed red blood cells in group 1 and 7 in group 2. Drainage related relevant SSI no significant difference has been proven.
As this study could not demonstrate a reduction of severe postoperative complications when using surgical drains, there is no indication for prophylactic subfascial closed-suction drains after musculoskeletal hip surgery in children.

***Poster-Presentation 14.03. 15.03. P.32***

### *Ulnar Longitudinal Deficiency in Patients with Hereditary Multiple Exostosis*

Bayraktar, V.*[1], Grifka, J.[1], Renkawitz, T.[1], Matussek, J.[1]

[1]Orthopädische Klinik der Universität Regensburg, Kinderorthopädie, Bad Abbach, Deutschland

***Question*** Ulnar longitudinal deficiency (ULD, also known as ulnar clubhand) is a very rare postaxial deformity and appears congenital or associated with a number of medical syndromes. We introduce 12 patients with ULD in hereditary multiple exostoses (HME), a skeletal disorder which primarily affects enchondral bone during growth. We performed in these patients a monoxial gradual distraction of the ulna followed by centralization. No recurrence of deformity were observed. To our knowledge this context with ULD in patients with HME has not been reported in literature.
***Objective*** Siehe oben.
***Methodology*** Siehe oben.
***Result*** Siehe oben.

***Poster-Presentation 14.03. 15.03. P.33***

### *Percutaneous Release of Trigger Thumb in Children: Surgical Technique, Review of the Literature and Presentation of 6 Own Cases*

Tedeus, M.*[1], Lamprecht, E.[1]

[1]Kantonsspital Winterthur, Kinderorthopädie, Winterthur, Schweiz

***Question*** Trigger digit in children has a reported incidence of 0.05–0.3 %. Most of the cases are diagnosed within the first 3 years of life. Predominantly, the thumb is affected. Often there is no triggering, but the finger is fixed in flexion.
Therapy includes physiotherapy and splinting especially in younger children under 1 year of age, and if the trigger finger persists, surgical release of the A1 pulley is performed.
The standard surgical procedure of trigger fingers—in children as well as in adults—is an open pulley release, which has good success rates and low complication rate. But nevertheless, some authors have reported side effects of open pulley release in children such as loss of IP joint motion and MCP hyperextension. As a reason, excessive release of the pulley is suspected.

In adults, percutaneous A1 pulley release has proven to be a promising alternative to open surgery: There is less risk of scar contraction and excessive pulley release, whereas the effectiveness of the percutaneous procedure is found to be equal to the open technique. Nevertheless, some authors have reported a higher risk of digital nerve injury.

Recently, the percutaneuous technique has been adapted for trigger finger release in children, and some authors are reporting very good results with no cases of digital nerve injury.

***Objective*** We want to give an overview of treatment options for trigger thumb in children and present the percutaneous surgical treatment option.

***Methodology*** We are presenting the surgical technique of percutaneous trigger thumb release in children and an overview of the literature along with six own cases.

***Result*** In all of our cases, trigger fingers could be successfully treated percutaneously with no adverse events and very good clinical outcome.

Open Access

The abstracts are distributed under the terms of the Creative Commons Attribution License which permits any use, distribution, and reproduction in any medium, provided the original author(s) and the source are credited.

